# How “pro” are probiotics for wildlife species? Novel data, lack of evidence, and future directions

**DOI:** 10.1093/ismeco/ycag036

**Published:** 2026-02-28

**Authors:** Sally L Bornbusch, Piper R Thacher, Marlitza Francisque, Alexandra L DeCandia, Robyn Bortner, Della Garelle, Erin L Kendrick, Michael T Maslanka, Carly R Muletz-Wolz

**Affiliations:** Smithsonian's National Zoo and Conservation Biology Institute, Center for Conservation Genomics, Washington, DC 20008, United States; Smithsonian's National Zoo and Conservation Biology Institute, Department of Nutrition Science, Washington, DC 20008, United States; Smithsonian's National Zoo and Conservation Biology Institute, Center for Conservation Genomics, Washington, DC 20008, United States; George Mason University, Smithsonian-Mason School of Conservation, Department of Environmental Science and Policy, Fairfax, VA 22030, United States; Pennsylvania State University, Department of Biomedical Engineering, University Park, PA 16802, United States; Smithsonian's National Zoo and Conservation Biology Institute, Center for Conservation Genomics, Washington, DC 20008, United States; Georgetown University, Department of Biology, Washington, DC 20057, United States; U.S. Fish and Wildlife Service, National Black-Footed Ferret Conservation Center, Carr, CO 80612, United States; U.S. Fish and Wildlife Service, National Black-Footed Ferret Conservation Center, Carr, CO 80612, United States; Smithsonian's National Zoo and Conservation Biology Institute, Department of Nutrition Science, Washington, DC 20008, United States; Smithsonian's National Zoo and Conservation Biology Institute, Department of Nutrition Science, Washington, DC 20008, United States; Smithsonian's National Zoo and Conservation Biology Institute, Center for Conservation Genomics, Washington, DC 20008, United States; National Institute for Standards and Technology, Complex Microbial Systems Group, Gaithersburg, MD 20899, United States

**Keywords:** commercial probiotics, animal microbiomes, microbial therapies, conservation, husbandry, endangered species, wildlife, black-footed ferrets, applied microbial ecology

## Abstract

Treatments that aim to purposefully manipulate host-associated microbiomes are now prevalent in human and animal medicine. Probiotics that contain live bacteria are purported to improve microbiome function and host health. Although research is advancing, commercial probiotic development has outpaced empirical study of probiotic efficacy. Probiotics are widely used in *ex-situ* wildlife care despite a lack of empirical study or support. We interrogate the relevance of commercial probiotics in *ex-situ* wildlife by (a) sequencing the composition of commercial probiotics used to treat wildlife, (b) comparing the probiotic sequences to data on the microbiomes of >900 animal species, and (c) characterizing the effects of a commercial probiotic on probiotic colonization, prevalence of a potential enteric pathogen (*Clostridium perfringens*), and metagenomic function in endangered black-footed ferrets (*Mustela nigripes*). We found mislabeling and potential contaminants in probiotics marketed for a range of species. The probiotic bacteria were rare or absent in published animal microbiomes. In black-footed ferrets, probiotic treatment induced minimal probiotic colonization, negligible functional change, and limited influence on the potential enteric pathogen. Given our findings, which reiterate concerns about the efficacy of commercial probiotics across human and animal sectors, greater effort must be put towards identifying species-specific probiotic candidates and studying alternative microbial therapies for wildlife under human care.

## Introduction

The development of treatments that purposefully modulate host-associated microbiomes has accelerated dramatically, capitalizing on scientific and public awareness of microbiomes. Probiotics are increasingly marketed in products for humans and domesticated animals as supplements and in foods [1–3]. Recently, probiotics have been incorporated into the care of wildlife species at zoos and through conservation efforts [4–7], despite limited empirical research on commercial probiotic effects in wildlife species. We suggest that the production and marketing of commercial probiotics, particularly for wildlife species, has outpaced empirical, safety and efficacy research. Given the variation in microbial structure and function across animal species [8–13], probiotics should be studied within microbial ecology frameworks and in the context of the recipient’s ecological, physiological, and microbial requirements.

To underscore the need for probiotic research in non-model animals, we provide brief context on commercial probiotic production. The definition of probiotics by the World Health Organization and the Food and Agriculture Organization of the United Nations is: “live microorganisms that, when administered in adequate amounts, confer a health benefit on the host” [14]. In the USA, oversight of commercial probiotics falls under the purview of the Food and Drug Administration (FDA), which defines probiotics as “direct-fed microbial products that are purported to contain live (viable) microorganisms (bacteria and/or yeast)” (defined in 1988, updated in 1995; [15–17]). The FDA allows manufacturers to market probiotics as dietary supplements, which undergo minimal safety testing and are only subject to post-market FDA regulation. There is no requirement that probiotic supplements be effective at influencing host health nor be tested in the target host species. This has allowed for extensive production of commercial probiotics, most of which lack empirical evidence supporting their use in wildlife and other animal species.

Extensive study of human microbiomes has identified probiotic species, such as *Lactobacillus* and *Bifidobacterium* members, that are considered beneficial [18, 19] and are now used in animal probiotics either in single- or multi-strain probiotic products [1, 20–24]. *Lactobacillus* and *Bifidobacterium*, along with other single- and multi-strain probiotic products, have been used for a wide range of health and productivity outcomes in agricultural and companion animals, including the treatment of gastrointestinal (GI) concerns and enteric infections [3, 25–28]. Bacteria in animal microbiomes are host-specific [29–31] and the interactions between probiotic species and an animals’ native microbiome will differ by host species and physiology. For example, *Lactobacillus* probiotics are considered beneficial in humans and, when used appropriately, can help maintain GI health in livestock [32, 33]. However, overabundances of lactobacilli in ruminants, combined with high-grain diets rich in fermentable carbohydrates, can cause an over-production of lactic acid and lead to potentially fatal ruminal acidosis [34, 35]. This highlights the importance of considering probiotics within the ecological and physiological contexts of the host species.

Identifying probiotics by mining host-specific microbial data, isolating, and preserving microbial candidates is an ecologically grounded approach that can yield probiotics that successfully promote health in the target host species [5]. Notable examples in wildlife include probiotic treatments for diseases such as chytridiomycosis in amphibians [36–39], white-nose syndrome in bats [40–42], snake fungal disease [43], and multiple pathogens and parasites in invertebrates such as corals [44, 45] and bees [46–48]. Nevertheless, most wildlife-care facilities lack the scientific capacity for developing targeted probiotics, instead relying on accessible commercial probiotics to treat animals. A primary reason for the use of commercial probiotics in *ex-situ* wildlife is the high prevalence of GI issues (e.g. *Clostridium* and *Helicobacter* spp., gastroenteritis), which mirror diseases seen in humans. Certain wildlife species and individuals are more vulnerable to GI afflictions. Black-footed ferrets (*Mustela nigripes*; BFFs or ferrets, throughout), e.g. are endangered, obligate carnivores that are susceptible to GI distress and infection, with *Clostridium perfringens* being a cause of morbidity and mortality in *ex-situ* BFFs [49–52]. Previous research has demonstrated that *C. perfringens* is present and, in some cases, abundant among the microbiomes of clinically healthy *ex-situ* BFFs [53], indicating that it is a native gut bacterium. Although acutely pathogenic *C. perfringens* infections are characterized by enterotoxin production, it is unclear why or how *C. perfringens* shifts from commensal to pathogenic [54]. There is thus a need for microbial therapies with the specific aim of improving GI health in *ex-situ* BFFs.

Our goal for this study was to assess probiotic products and practices being used in the care of diverse *ex-situ* wildlife. Given the regulation and rapid production of commercial probiotics, we hypothesized that probiotics used in wildlife care would not be congruent with wildlife microbial ecology. To test this hypothesis, we first identified commercial probiotics actively being used to treat wildlife species at US zoos and performed 16S rRNA amplicon sequencing to assess probiotic membership. We then compared the commercial probiotic sequences to publicly available gut microbiome data for >900 wildlife species [55]. Given the host specificity of gut bacteria, we expected that the commercial probiotic microbes would not be abundant in animal communities. Finally, to test the influence of a commercial probiotic in wildlife, we performed a control–treatment trial of a prophylactic probiotic in BFFs, using 16S rRNA amplicon and shotgun metagenomic sequencing to examine rates of colonization, changes in *C. perfringens* prevalence, bacterial network associations, and variation in microbial function. Given anecdotal indication that this specific commercial probiotic lowered BFF morbidity, we expected moderate colonization of probiotic microbes and shifts in microbial function reflecting health-promoting functional pathways.

## Materials and methods

### Probiotic identification and processing

We surveyed 14 US zoos on whether commercial probiotics were used in the care of their animal and, if so, which products were used. From this survey, we identified 11 probiotics that we actively being used in zoo wildlife species and purchased the 11 probiotics from the manufacturers. We prepared the probiotics for DNA extraction by homogenizing 1 g of probiotic material with 250 μl of UV-sterilized, ultrapure type 1 MilliQ Q-POD® water. The probiotics were extracted in random order, in quadruplicate, and all probiotics were extracted, prepared, and sequenced in one batch (see Supplementary Information for additional probiotic handling and processing information).

### Ferret experimental design and sample collection

Study subjects included 24 healthy BFFs housed at the US Fish and Wildlife National Black-footed Ferret Conservation Center (FCC) (Supplementary Table S1; see Supplementary Information for details on BFF husbandry). BFFs were split into two experimental groups with relatively even sex ratios and age ranges (Supplementary Table S1): Controls (CON, *n* = 12) and probiotic-treated animals (PBX; *n* = 12). For probiotic treatment, we selected a multi-strain probiotic that had previously been used prophylactically in FCC BFFs, with anecdotal suggestion that it decreased incidences of GI distress. But the probiotic had never been empirically tested in BFFs. The probiotic was labeled with eight bacterial species—six *Lactobacillus* and two *Bifidobacterium* (see Supplementary Table S2 for complete list of probiotic ingredients). For PBX animals, we followed the manufacturer’s instructions and mixed ~0.5 g of probiotic powder into the diet daily for 7 days during Week 3 of the experiment (W3, Day 0–6) and animal caretakers monitored to ensure the diet was completely consumed.

Fresh fecal samples (<4 h post defecation) were collected from all ferrets twice weekly before, during, and after the probiotic trial, spanning 6 weeks (W1 to W6). Fecal samples were collected in sterile tubes and stored at −80°C until being shipped on dry ice to the Center for Conservation Genomics at the Smithsonian’s National Zoo and Conservation Biology Institute. All animal protocols were approved by the US Fish and Wildlife BFF Recovery Program and the National BFF Conservation Center IACUC committee (NBFFCC IACUC # 2022-4).

### Preparation and sequencing of probiotic and ferret samples

DNA extractions of probiotic and ferret samples were performed using the DNeasy PowerSoil Pro QIAcube HT kit following the manufacturer’s protocol (QIAGEN, Germany). We additionally extracted negative controls (empty tubes) and positive controls (ZymoBIOMICS microbial community; Zymo, Irvine, CA, USA; Catalog No. D6300). For 16S rRNA amplicon sequencing of all samples, we prepared DNA libraries for the V4 to V5 region (515F-Y and 939R primers) using a previously published protocol [53] and sequenced on an Illumina MiSeq run (2 × 300 bp paired-end reads). For shotgun metagenomic sampling, extracted DNA from a subset of ferret samples (*n* = 72 samples; CON = 9 ferrets, PBX, *n* = 9 ferrets, on Days −11, 3, 9, and 15 corresponding to W1, W3, W4, and W5) was sent to the Oklahoma Medical Research Foundation for library preparation and sequencing on the Illumina NovaSeq platform. See Supplementary Information for details on amplicon and shotgun library preparation methods.

### 16S amplicon bioinformatics

We used QIIME2 (v. 2023.9) [56] and RStudio (R v. 4.3.2) [57] to process the demultiplexed sequence data. Briefly, we used dada2 in QIIME2 to quality filter and trim sequences, merge forward and reverse reads, remove chimeric sequences, and generate amplicon sequence variants (ASVs) [58]. We assigned taxonomy using a Naïve Bayes classifier pre-trained on SILVA v. 138.1 99% full-length sequences. We applied the NCBI BLASTn tool to query representative sequences of analytical interest against NCBI GenBank’s 16S rRNA sequence database. Using the decontam package in R [59], we removed 51 potential contaminant ASVs [60]. See Supplementary Information for additional filtering and assessment of spurious ASVs.

We identified potential contaminant ASVs in the commercial probiotics as ASVs that were (a) not present in our negative or positive controls, (b) not removed via decontam, (c) identified in all four replicates of a given probiotic but not present in all 44 probiotic samples (i.e. further ruling out contamination from laboratory protocols or reagents), and (d) not assigned to a genus listed in the probiotic ingredients. An ASV must have matched all four criteria to be considered a contaminant.

We used Geneious to compare sequences from the commercial probiotics to sequences from a publicly available dataset by on the gut microbiomes of >900 animal species [55]. Given that only our forward primers matched those used in the Song *et al*. dataset, we only compared forward reads. Additionally, because the Song *et al*. sequences were trimmed to 100 bp whereas our sequences were over 300 bp, we only considered matching sequences that aligned at the forward end and matched with 100% sequence identity for the full 100 bp length.

### Metagenomic bioinformatics

Raw sequence data were processed using a read-based mapping approach. We first used bowtie2 [60] to remove sequences that mapped to the BFF genome [61]. We used fastp [62] to trim and quality filter reads, trimming Illumina adapters and removing sequences with fewer than 50 reads or phred quality scores <20. Resulting filtered data included a total of ~1.3 billion reads across 72 samples, with an average depth of ~18.6 million reads.

To assign taxonomic identity, we used MetaPhlAn4 (v. 4.1) [63] on concatenated forward and reverse reads, using marker genes identified across microbial whole genomes and metagenome-assembled genomes and grouping into species-level genome bins. We used HUMAnN3 to identify functional gene pathways by mapping sequence reads to pangenomes using bowtie2 and the ChocoPhlAn database and subsequently aligning unmapped reads to non-organismal protein databases with DIAMOND and UniRef90 database [64]. Gene families and pathway abundances were, by default, normalized for gene length (i.e. reads per kilobase), and were normalized for sequencing depth by transforming abundances to copies per million (cpm).

### Statistics

To characterize the influence of probiotic treatment on ferret gut microbiomes, we focused on the amplicon data—with greater temporal resolution—for longitudinal analyses and used the metagenomic data to assess variation between the two experimental groups at Days −11, 3, 9, and 15 in relation to probiotic treatment.

For the amplicon data, we calculated measures of bacterial diversity (ASV richness, Shannon diversity, and Faith’s phylogenetic diversity) and bacterial composition (Bray-Curtis, Jaccard, and generalized UniFrac (GUF)). We visualized bacterial composition using Principal Coordinate Analysis and tested for variation using PERMANOVAs with experimental group and day as fixed effects and animal as a random effect (adonis2, R). To account for compositionality, we applied centered log-ratio (CLR) transformations to raw sequence counts. To assess variation in bacterial diversity and CLR-transformed abundances, we used hierarchical generalized additive models (HGAMs) across control and treated animals [65]. Our HGAM included terms for group-specific smoothers for each treatment that account for variable response trajectory over time, group-specific intercepts that represent random effects for different treatment intercepts, and random effects for the intercepts of each animal. Smoothing terms that are found to be significant indicate longitudinal trajectories that differ significantly from the null hypothesis of a flat, linear relationship.

To characterize associations between bacterial ASVs, we constructed seven association networks for each experimental group: one that included all samples for the respective group, and one for each of the 6 sampling weeks (W1–W6). We used Network Construction and Comparison for Microbiome Data (NetCoMi) [66], with CLR normalization, to construct association networks and compare networks between experimental groups. Using these association networks, we further performed differential association analysis and generated differential networks to identify and visualize pairs of taxa that had differential associations across multiple networks. See Supplementary Information for additional details on network construction.

For the shotgun metagenomic data, we calculated two measures of functional diversity (Shannon and Simpson diversity) and two measures of functional composition (Bray-Curtis and Jaccard). We used linear mixed-effect models (LMMs) for strain-level taxonomic and functional diversity and PERMANOVAs of composition to assess variation between treatment groups at specific timepoints. We additionally used MaAsLin 3—microbiome multivariable associations with linear models—to assess variation in functional pathways between the two treatment groups across all four timepoints [67]. Our model formula included group and week as fixed effects with animal as a random effect to account for repeat sampling. We further analyzed variation in functional pathway abundance within each timepoint using linear models with group as a fixed effect.

## Results

### Commercial probiotic membership

**Figure 1:**
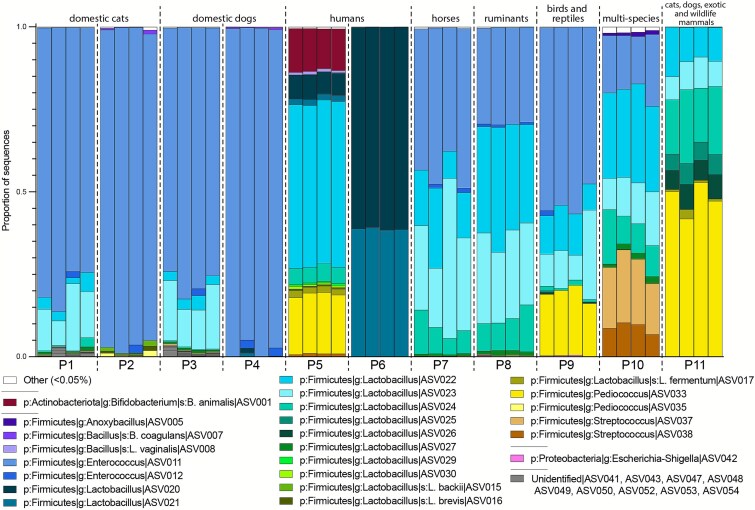
Relative abundances of ASVs with greater than 0.5% of reads in 11 commercial probiotics (P1–P11), sequenced in replicates of four. Colors denote individual ASVs, except for nine ASVs that could not be identified beyond the kingdom bacteria (in gray). For labeled ingredients in each probiotic, see Table 1.

The 11 probiotics were labeled for use in a wide variety of domestic animals and “wildlife” (Table 1). The labels listed between 1 and 20 bacterial strains, encompassing 24 taxa from 5 genera: *Lactobacillus, Bifidobacterium, Pediococcus, Streptococcus*, and *Enterococcus* (Table 1). *Enterococcus faecium* was labeled in 9 out of 11 probiotics and at least one *Lactobacillus* sp. was labeled in 9 out of 11 probiotics. Five of the 11 probiotics (P1, P3, P7, P8, and P10) were manufactured by the same company, labeled for respective use in cats, dogs, horses, ruminants, and “multi-species,” and labeled to have the same four microbial members (Table 1). Among these five probiotics: (a) P1 for cats and P3 for dogs had similar membership, dominated by a single *Enterococcus* ASV, (b) P7 for horses and P8 for ruminants also had similar membership with more equitable representation of *Enterococcus* and *Lactobacillus* ASVs, and (c) P10 for “multi-species” had distinct membership. The two probiotics labeled for use in humans—P5 and P6—showed the least variation in membership among their respective replicates (Fig. 1).

**Table 1:** List of analyzed commercial probiotics (P1–P11) with their target species, and the number and identity of taxa listed on the label.

**Probiotic (P#)**	**Target**	**# Taxa on label**	**Strains**
P1^*^	Cats	4	*Enterococcus faecium, Lactobacillus acidophilus, Lactobacillus plantarum, Lactobacillus casei*
P2^**^	Cats	1	*E. faecium*
P3^*^	Dogs	4	*E. faecium, L. acidophilus, L. plantarum, L. casei*
P4^**^	Dogs	1	*E. faecium*
P5	Humans	20	*L. acidophilus, Lactobacillus rhamnosus, Lactobacillus crispatus, L. plantarum, Lactobacillus paracasei, Lactobacillus bulgaricus, Lactobacillus reuteri, L. casei, Lactobacillus salivarius, Lactobacillus helveticus, Lactobacillus gasseri, Bifidobacterium lactis, Bifidobacterium breve, Bifidobacterium adolescentis, Bifidobacterium infantis, Bifidobacterium Longum, Lactococcus lactis, Pediococcus pentosaceus, Pediococcus acidilactici, Streptococcus thermophiles*
P6	Humans	1	*L. rhamnosus*
P7^*^	Horses	4	*E. faecium, L. acidophilus, L. plantarum, L. casei*
P8^*^	ruminants	4	*E. faecium, L. acidophilus, L. plantarum, L. casei*
P9^***^	Birds and reptiles	5	*Lactobacillus fermentum, Lactobacillus casei* (avian strain), *Lactobacillus plantarum, Lactobacillus acidophilus, Streptococcus faecium*
P10^*^	Multi-species	4	*E. faecium, L. acidophilus, L. plantarum, L. casei*
P11^***^	Cats, dogs, exotic and wildlife mammals	8	*Bifidobacterium bifidum, Lactobacillus fermentum, L. acidophilus, Lactobacillus casei, L. plantarum, E. faecium, B. bifidum, Pediococcus acidilactici*

Asterisks denote probiotics made by the same manufacturer (^*^Manufacturer #1, etc.).

We found evidence of 15 potential contaminant ASVs or mislabeled taxa across seven probiotics (Table 2). Although generally rare, contaminant relative abundance ranged from <0.01% to 22.1% within a given sample. P10 included two *Streptococcus* ASVs (Fig. 1), with ASV37 being highly abundant (6.77% to 22.10%), despite not listing any members of the *Streptococcus* genus on the label. *Streptococcus* ASV37 was also found at low abundances in three probiotics made by the same manufacturer as P10 (P1, P3, and P8). In P9, we identified a *Pediococcus* ASV with abundances between 16.17% and 21.18%, despite the label not including *Pediococcus* members (Fig. 1). P9 was manufactured by the same company as P11, with P11 labeled to include *Pediococcus* and harboring the same *Pediococcus* ASV in high abundances. P9 further listed *Streptococcus faecium* on its label, but we found no *Streptococcus* sequences. We did find an *Enterococcus* ASV that is likely *Enterococcus faecium*. Although *E. faecium* was previously placed in the *Streptococcus* genus, it had long previously been reassigned to the *Enterococcus* genus, suggesting potential mislabeling of this taxon.

**Table 2:** List of potential contaminant ASVs, with taxonomy identification, range of relative abundance (percent of sequences) across all probiotic samples, and the probiotics in which the ASV is considered a contaminant.

**ASV**	**Taxonomy**	**Abundance**	**Probiotics**
ASV003	g_*Cutibacterium* | ASV003	0.01% to 0.11%	P7, P8
ASV004	g_*Thermus* | s_*Thermus_thermophilus* | ASV004	0.10% to 0.22%	P10
ASV005	g_*Anoxybacillus* | ASV005	0.75% to 1.28%	P10
ASV007	g_*Bacillus* | s_*Bacillus_coagulans* | ASV007	0.11% to 1.15%	P2, P4
ASV010	g_*Geobacillus* | ASV010	0.14% to 0.20%	P10
ASV015	g_*Lactobacillus* | s_*Lactobacillus_backii* | ASV015	0.35% to 1.75%	P2
ASV016	g_*Lactobacillus* | s_*Lactobacillus_brevis* | ASV016	0.29% to 1.26%	P2
ASV035	g_*Pediococcus* | ASV035	0.4% to 1.95%	P9
ASV042	g_*Escherichia Shigella* | ASV042	0.01% to 0.56%	P7, P8, P9, P11
ASV037	g_*Streptococcus* | ASV037	0.22% to 22.1%	P3, P10
ASV038	g_*Streptococcus* | ASV038	0.09% to 10.37%	P1, P3, P8, P10
ASV039	g_*Streptococcus* | ASV039	0.01% to 0.31%	P1, P3
ASV047	Unidentified | ASV047	0.05% to 0.26%	P1, P3
ASV048	Unidentified | ASV048	0.21% to 0.98%	P1, P3
ASV049	Unidentified | ASV049	0.37% to 1.68%	P1, P3

Contaminant ASVs are those that fulfilled all of four criteria: (a) not present in our negative or positive controls, (b) not removed via decontam, (c) identified in all four replicates of a given probiotic but were not present in all 44 probiotic samples, and (d) not assigned to a genus listed in the probiotic ingredients.

### Comparison of probiotic taxa to animal gut microbiomes

When comparing probiotic sequences to 16S rRNA data across animal species, we found that most probiotic taxa were rare or absent across host species. Comparing our probiotic sequences to the Song *et al*. dataset [55] of gut microbiomes from >900 vertebrate species—22 707 representative sequences and 6141 samples—we identified eight representative sequences that fully matched to probiotic sequences: *Lactobacillus* (four sequences), *Enterococcus* (1), *Streptococcus* (1), *Bifidobacterium* (1), and Lactobacillaceae (1) (Supplementary Table S3). In the Song *et al*. dataset, these eight sequences represented taxa with total read counts from 30 to 395 675. The *Enterococcus* sequence was present in 1680 animal samples, spanning 605 host species. This same *Enterococcus* taxon was the most prevalent within animal guts, accounting for upwards of 99% of reads in certain species (spotted free-tailed bat [*Chaerephon bivitattus*] and silver-beaked tanager [*Ramphocelus carbo*]), although other studies of these species’ microbiomes do not show *Enterococcus* dominance [68, 69].

### Influence of commercial probiotic treatment on diversity and composition of ferret gut microbiomes

**Figure 2:**
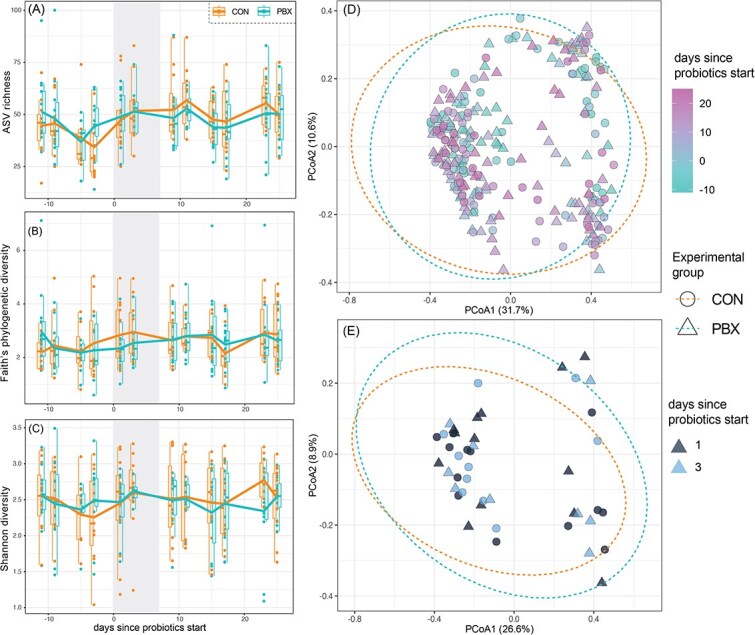
Alpha and beta diversity in microbiomes of black-footed ferrets in CON or PBX experimental groups. (A) ASVs richness, (B) Faith’s phylogenetic diversity, and (C) Shannon diversity over time, with the vertical gray bar representing the period of probiotic treatment (Day 0 to Day 6). Principal coordinate analyses of Bray-Curtis distance for (A) all samples and (B) during the period of probiotic treatment.

When examining the diversity and composition of ferret microbiomes using amplicon data, we found little variation according to treatment. During the treatment period, no measure of bacterial diversity varied between CON and PBX animals (Shannon diversity: *t* = 0.09, *P* = .93; ASV richness: *t* = −0.21, *P* = .83; Faith’s phylogenetic diversity: *t* = −1.78, *P* = .08; Fig. 2). When examining longitudinal trajectories across the entire study period, observed ASVs and phylogenetic diversity both varied significantly over time, but only in the CON group (Supplementary Table S4 HGAMs). There was also significant inter-individual variation in bacterial diversity within both treatment groups (Supplementary Table S4). When examining composition of the microbiome (beta diversity), we found no significant difference between group or over time (PERMANOVA: all *P* > .05; Bray-Curtis: *R*^2^ = 0.002, *F* = 0.76; Jaccard: *R*^2^ = 0.003, *F* = 0.89; GUF: *R*^2^ = 0.002, *F* = 0.70), nor during the treatment period (PERMANOVA: all *P* > .05; Bray-Curtis: *R*^2^ = 0.014, *F* = 0.61; Jaccard: *R*^2^ = 0.015, *F* = 0.66; GUF: *R*^2^ = 0.018, *F* = 0.80).

### Probiotic colonization—*Lactobacillus* and *Bifidobacterium*—in ferret microbiomes

**Figure 3:**
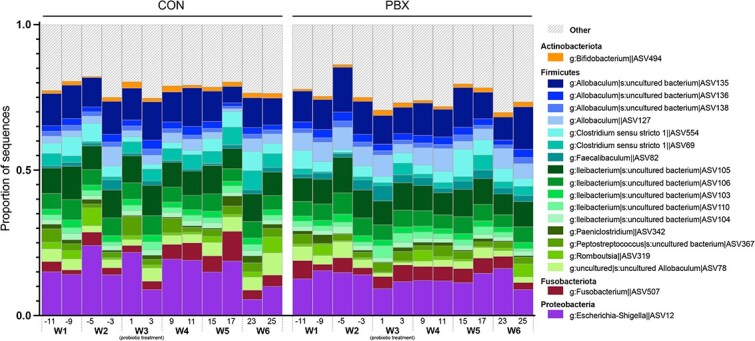
Relative abundances of ASVs in the gut microbiomes of black-footed ferrets in CON or PBX experimental groups. Colors denote individual ASVs, and the category other denotes a conglomerate of rare taxa (representing <1% of sequences). The *x*-axis represents the number of days since the start of probiotic treatment and the week (W1–W6), with the first dose of probiotic on Day 0 and the final dose on Day 6, spanning Week 3 (W3).

**Figure 4:**
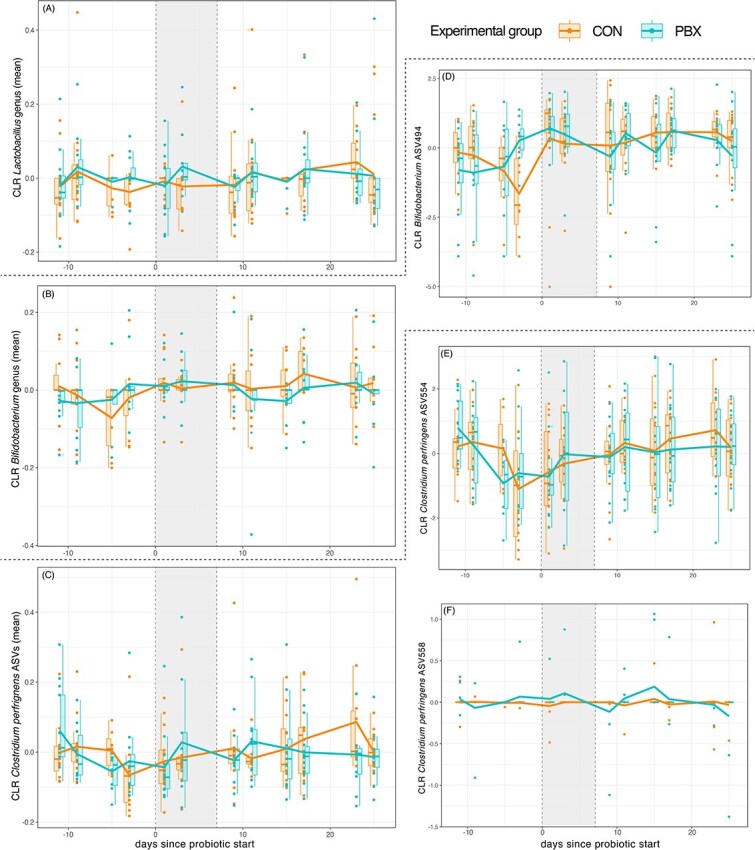
CLR transformed abundances of bacterial taxa in the microbiomes of black-footed ferrets in CON or PBX experimental groups. (A) Genus *Lactobacillus*, (B) genus *Bifidobacterium*, (C) combined *Clostridium perfringens* ASVs, and (D–F) specific ASVs with significant variation in longitudinal trajectories. The vertical gray bar represents the period of probiotic treatment (Day 0 to Day 6).

We found little evidence of probiotic colonization in BFF microbiomes using both amplicon and shotgun metagenomic sequencing (Fig. 3). When testing for colonization of probiotic taxa in the amplicon data, we found 45 ASVs that were identified as either *Lactobacillus* (33 ASVs) or *Bifidobacterium* (12 ASVs) (Fig. 4 and Supplementary Fig. S2). None of the *Lactobacillus* nor *Bifidobacterium* ASVs were present in every sample. During the treatment period, none of the 27 ASVs (21 *Lactobacillus*, 6 *Bifidobacterium*) that had non-zero CLR-transformed abundances varied significantly in abundance between the CON and PBX group (LMMs; Table 3 and Supplementary Fig. S1). When examining longitudinal trajectories at the genus level, the CLR abundances of *Lactobacillus* and *Bifidobacterium* showed no significant variation in trajectory (HGAMs; Fig. 4A and B). At the ASV level, three *Bifidobacterium* ASVs showed significant variation over time in the CON group, while one showed variation in the PBX group, reflecting lower abundance before probiotic treatment (Supplementary Table S5 and Fig. 4D). One *Lactobacillus* ASV showed significant variation over time in the CON group, while two varied in the PBX group (Supplementary Table S5 and Supplementary Fig. S1), likely reflecting outlier samples occurring at the beginning and end of the study (Supplementary Fig. S1).

**Table 3:** Results of linear models of CLR transformed abundances of genera and ASVs assigned to *Bifidobacterium, Lactobacillus*, or *Clostridium sensu stricto 1*.

**Taxonomy**	**ASV ID**	**Estimate**	**Std. Error**	***t* value**	***P*-value**
*Bifidobacterium* genus mean		0.003	0.018	0.179	.858
*Bifidobacterium*	ASV494	0.355	0.417	0.851	.399
*Bifidobacterium*	ASV495	−0.017	0.011	−1.599	.117
*Bifidobacterium*	ASV496	0.000	0.000	NA	NA
*Bifidobacterium (Bifidobacterium_animalis)*	ASV497	−0.049	0.036	−1.372	.177
*Bifidobacterium*	ASV498	0.140	0.169	0.829	.412
*Bifidobacterium (Bifidobacterium_bifidum)*	ASV502	−0.042	0.041	−1.023	.312
*Bifidobacterium*	ASV504	0.000	0.000	NA	NA
*Lactobacillus* genus mean		0.015	0.024	0.618	.540
*Lactobacillus*	ASV175	0.366	0.236	1.551	.128
*Lactobacillus*	ASV177	0.000	0.000	NA	NA
*Lactobacillus*	ASV178	0.000	0.000	NA	NA
*Lactobacillus*	ASV179	0.038	0.032	1.213	.232
*Lactobacillus*	ASV182	−0.014	0.089	−0.155	.878
*Lactobacillus*	ASV183	0.000	0.000	NA	NA
*Lactobacillus (Lactobacillus_saerimneri)*	ASV184	−0.056	0.055	−1.023	.312
*Lactobacillus*	ASV187	−0.181	0.199	−0.907	.370
*Lactobacillus*	ASV188	0.000	0.000	NA	NA
*Lactobacillus*	ASV189	0.000	0.000	NA	NA
*Lactobacillus*	ASV191	−0.121	0.316	−0.383	.704
*Lactobacillus (Lactobacillus_intestinalis)*	ASV194	0.441	0.339	1.303	.200
*Lactobacillus*	ASV195	0.000	0.000	NA	NA
*Lactobacillus*	ASV196	0.000	0.000	NA	NA
*Lactobacillus (Lactobacillus_intestinalis)*	ASV197	0.021	0.021	1.023	.312
*Lactobacillus (Lactobacillus_brevis)*	ASV198	0.041	0.052	0.785	.437
*Lactobacillus*	ASV199	−0.150	0.101	−1.478	.147
*Lactobacillus*	ASV200	0.198	0.235	0.841	.405
*Lactobacillus*	ASV201	−0.067	0.067	−1.003	.321
*Lactobacillus*	ASV203	0.000	0.027	0.000	1.000
*Lactobacillus (Lactobacillus_fermentum)*	ASV204	−0.018	0.017	−1.055	.297
*C. perfringens* mean		0.008	0.036	0.214	.832
*Clostridium_sensu_stricto_1*	ASV1	−0.034	0.037	−0.912	.367
*Clostridium_sensu_stricto_1*	ASV2	−0.025	0.028	−0.909	.368
*Clostridium_sensu_stricto_1*	ASV3	−0.006	0.008	−0.743	.462
*Clostridium_sensu_stricto_1*	ASV553	0.096	0.129	0.742	.462
*Clostridium_sensu_stricto_1*	ASV554	0.030	0.431	0.070	.945
*Clostridium_sensu_stricto_1*	ASV555	0.047	0.046	1.023	.312
*Clostridium_sensu_stricto_1*	ASV556	−0.047	0.048	−0.978	.334
*Clostridium_sensu_stricto_1*	ASV557	0.000	0.000	NA	NA
*Clostridium_sensu_stricto_1*	ASV558	0.094	0.049	1.895	.065
*Clostridium_sensu_stricto_1*	ASV559	0.128	0.087	1.472	.148
*Clostridium_sensu_stricto_1*	ASV560	−0.074	0.052	−1.411	.165
*Clostridium_sensu_stricto_1*	ASV561	−0.029	0.037	−0.784	.437
*Clostridium_sensu_stricto_1*	ASV562	−0.145	0.103	−1.415	.164
*Clostridium_sensu_stricto_1*	ASV563	−0.021	0.056	−0.375	.709
*Clostridium_sensu_stricto_1*	ASV564	0.000	0.000	NA	NA
*Clostridium_sensu_stricto_1*	ASV565	0.123	0.084	1.475	.147
*Clostridium_sensu_stricto_1*	ASV566	0.000	0.000	NA	NA
*Clostridium_sensu_stricto_1*	ASV567	0.000	0.000	NA	NA

ASVs assigned to *Clostridium sensu stricto 1*. We further identified as *Clostridium perfringens* using NCBI BLAST. “NA” values indicate taxa for which CLR values were namely zeros.

Using shotgun metagenomics to assess species- and strain-level variation, we identified three species of *Lactobacillus*, each with one strain (Supplementary Table S6). Only *Lactobacillus acidophilus* matched a species listed on the probiotic label (Supplementary Table S2). We further found seven species of *Bifidobacterium* encompassing eight strains (two of *B. animalis*) (Supplementary Table S6). *Bifidobacterium bifidum* and *Bifidobacterium longum* were listed on the probiotic label. Across all 12 *Lactobacillus* and *Bifidobacterium* strains, only one—*B. pseudolongum*, a species not on the probiotic label—showed mean relative abundance greater than 1% and none were present in all individuals (Supplementary Table S6 and Supplementary Fig. S2). None of the *Lactobacillus* and *Bifidobacterium* strains varied significantly between CON and PBX groups at any of the four timepoints (Supplementary Fig. S2).

### Probiotic influence on *C. perfringens* in ferret microbiomes

Probiotic treatment had minimal impact on the abundance of *C. perfringens* in BFF guts. Using amplicon data, we identified 18 ASVs that matched to *C. perfringens* using NCBI BLAST (>99% sequences identity, 100% query cover, *E*-value = 0). Although none of the 18 ASVs were present in every sample, ASV554 had high relative abundances (mean > 1%, max = 67.95%). During the treatment period, none of the *C. perfringens* ASVs varied significantly in abundance between the CON and PBX group (LMMs; Table 3 and Supplementary Fig. S3). The mean CLR abundance of all *C. perfringens* ASVs showed no significant variation during probiotic treatment (LMM, Supplementary Table S5) nor in overall longitudinal trajectories (HGAMs; Supplementary Table S5 and Fig. 4C). At the ASV level, we found that *C. perfringens* ASV554 had a significantly variable trajectory over time in both the CON and PBX groups (HGAMs), but with trajectories reflecting changes that occurred prior to PBX treatment (Fig. 4E and Supplementary Table S5). We additionally found that *C. perfringens* ASV558 had a significantly variable trajectory over time, but only in the PBX group and only in a few animals that had qualitatively greater abundances of this ASV compared to CON animals (Fig. 4F). Results from shotgun metagenomics identified one strain of *C. perfringens* (mean = 7.2%, max = 60.9%; Supplementary Table S6). However, the abundance of this *C. perfringens* strain did not vary between experimental groups at any of the four timepoints (Supplementary Fig. S2).

### Probiotic treatment and bacterial association networks in ferret microbiomes

**Figure 5:**
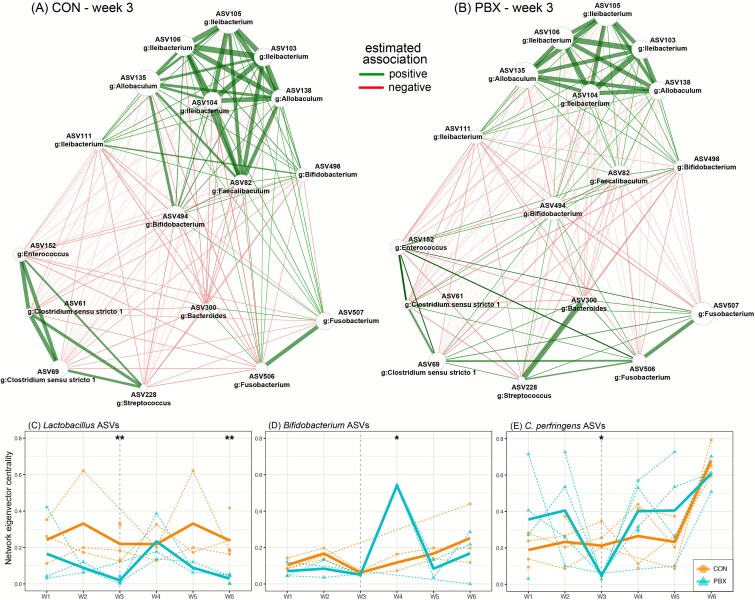
Differential association networks for the microbiomes of black-footed ferrets in (A) CON or (B) PBX experimental groups during the period of probiotic treatment (Week 3). Networks were constructed with center log-ratio normalized abundances of ASVs and differential pairwise associations were calculated using Fisher’s *z*-test. Edge weights are scaled to (non-negative) similarities. (C and D) Longitudinal variation in eigenvector centrality across networks calculated for each week of the study (W1–W6) for (C) *Lactobacillus*, (D) *Bifidobacterium*, and (E) *Clostridium perfringens* ASVs. Dashed colored lines are the trajectories of individuals ASVs, with the bold colored lines representing the mean of the ASVs within each genus, colored by experimental group. The vertical dashed line represents the week of probiotic treatment. ^*^P < .05, ^**^P < .01. ASVs within each genus, colored by experimental group. The vertical dashed line represents the week of probiotic treatment. ^*^P < .05, ^**^P < .01.

Bacterial associations of amplicon data varied within and between the CON and PBX groups over time. For the association networks that included all samples, *Clostridium sensu stricto 1* ASV70 was identified as a hub for both the CON and PBX group and was the only hub for the PBX group. The CON group had two additional hubs, *Lactobacillus* ASV182 and an ASV in family Lachnospiracaeae (Supplementary Fig. S4). In bacterial networks of the probiotic treatment period (W3), the CON network hubs included ASVs in the genera *Enterococcus* (ASV147), *Clostridium sensu stricto 1* (ASV57), and *Escherichia—Shigella* (ASV9), as well an ASV in family Erysipelotrichaceae. The PBX network hubs included ASVs in the genera *Clostridium sensu stricto 1* (ASV42) and *Ileibacterium* (ASV115 and ASV122) (Supplementary Fig. S4). Using differential network analysis, we identified 17 taxa—including 2 *Bifidobacterium* ASVs—comprising 88 pairwise associations that varied significantly between the CON and PBX networks during probiotic treatment (Fig. 5 and Supplementary Table S7). Notably, despite the number of positive associations being similar between the CON and PBX differential networks (Fig. 5; CON, *n* = 26, PBX, *n* = 29), the two *Bifidobacterium* ASVs had, on average, stronger positive associations in the CON network vs. the PBX network (CON mean association coefficient = 0.39 vs. PBX mean = 0.26). The number and strength of the *Bifidobacterium* negative associations was similar in both groups (CON, *n* = 34, mean = −0.24; PBX, *n* = 31, mean = −0.24).

Within the networks, potential probiotic taxa—*Lactobacillus* and *Bifidobacterium* ASVs—had varying associations between experimental groups and over time (Fig. 5). Eight *Lactobacillus* and three *Bifidobacterium* ASVs were included in at least one of the weekly association networks. When analyzing network metrics for the *Lactobacillus* ASVs across the 6 weeks, the interaction between group and week was non-significant for eigenvector centrality (Linear model (LM): *F* = 1.04, *P* = 0.41). During W3 and W6, however, *Lactobacillus* eigenvector centrality was significantly higher in the CON group compared to the PBX group (LM: W3, *t* = 6.67, *P* = .0001; W6, *t* = 7.05, *P* = .002). For the *Bifidobacterium* ASVs, the interaction between group and week was a significant predictor of eigenvector centrality (LM: *F* = 5.27, *P* = .005). The *Bifidobacterium* ASVs had greater eigenvector centrality in the PBX group during W4 (LM: *t* = 9.25, *P* = .01) but not during W3 (LM: *t* = −1.09 *P* = .38). The two *Bifidobacterium* ASVs driving this pattern were identified as *B. pseudolongum*, which was not a species list on the probiotic.

We found seven *C. perfringens* ASVs in at least one weekly association network. The interaction between group and week was non-significant for *C. perfringens* eigenvector centrality (LM: *F* = 1.39, *P* = .25). However, during probiotic treatment only (W3), the *C. perfringens* ASVs had lower eigenvector centrality in PBX compared to CON animals (LM: *F* = −2.57, *P* = .04), indicating the *C. perfringens* was temporarily less influential in the network of PBX animals during probiotic treatment.

### Probiotic influence on BFF microbiome function

**Figure 6:**
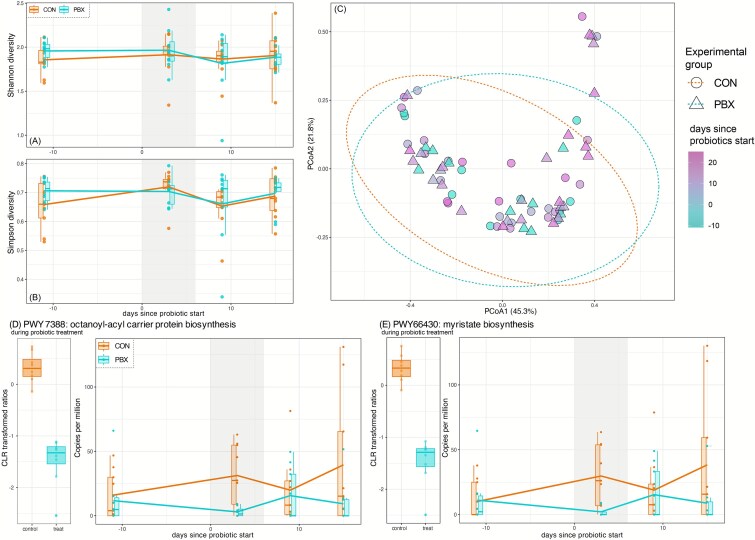
Functional diversity, composition, and pathway abundance for the microbiomes of black-footed ferrets in CON or PBX experimental groups. (A) Shannon diversity and (B) Simpson diversity of functional gene pathways over time, with the vertical gray bar representing the period of probiotic treatment (Day 0 to Day 6). (B) Principal coordinate analysis of Bray-Curtis distance of functional gene pathways, shaped according to experimental group and colored according to the number of days since the start of probiotic treatment. (D and E) Transformed abundance during probiotic treatment (CLR) and longitudinal abundance (cpm) for functional pathways found to vary significantly between experimental groups during the period of probiotic treatment (microbiome multivariable associations with linear models 3, MaAsLin3).

Metagenomic data showed minimal influence of probiotic treatment on ferret microbiome function. Across all samples, we identified 149 956 gene families (mean = 51 896), which grouped into 4425 metabolic pathways. The most abundant pathways included glycolysis (PWY-1042; mean = 816 cpm), peptidoglycan maturation (PWY-1586; mean = 677 cpm), and l-valine biosynthesis (VALSYN-PWY; 631 cpm). The diversity of functional pathways did not vary between groups nor over time (all *P*-values >.05; Fig. 6). Similarly, functional composition showed no significant variation according to treatment group, day, nor their interaction (all *P*-values >.05; Fig. 6).

When analyzing the trajectories of functional pathway abundance in the ferret microbiomes probiotic treatment, we found no significant variation in any pathway trajectory between the CON and PBX groups (Fig. 6). During the period of probiotic treatment, we found only two pathways that varied in abundance—but not prevalence—between CON and PBX ferrets: Octanoyl-acyl carrier protein biosynthesis (PWY 7388) in *E. coli* (MaAsLin3: *q* = 0.013, *P* < .0001) and myristate biosynthesis (PWY66–430) also in *E. coli* (MaAsLin3: *q* = 0.014, *P* < .0001), both of which showed greater abundance in CON animals compared to PBX animals (Fig. 6). Notably, this pattern was driven by greater abundance of these two pathways in CON animals, whereas the abundances in PBX animals showed little variation.

## Discussion

Here, we investigated the bacterial composition of multiple commercial probiotics, analyzed the prevalence of probiotic bacteria in wildlife gut microbiomes, and assessed the impact of a commercial probiotic on gut microbiome structure and function in *ex-situ* endangered wildlife. We found discrepancies in probiotic membership, including potential contaminants, reinforcing previous studies of commercial probiotics [70]. The probiotic taxa were largely absent from wildlife gut microbiomes, with exceptions being *Enterococcus* spp. Commercial probiotics had minimal effect on BFF gut microbiomes, with some evidence in the bacterial association networks but negligible impact on microbiome function. Although sparse, previous studies on commercial probiotic treatment in wildlife have found mixed results, with some showing shifted microbiota and improved health metrics and others showing minimal or no influence on microbiota and host health [6, 71–73]. Overall, evidence supporting the relevance of commercial probiotics to wildlife microbiomes is limited, emphasizing the need to critically consider probiotics through the lens of applied wildlife care.

Across the 11 commercial probiotics, microbial membership did not seem to reflect the target host species. Five probiotics produced by the same manufacturer but marketed for a range of animals, including “wildlife,” were labeled with the same four microbial constituents. However, the bacterial proportions were not equitable across these probiotics with the same label, and it was unclear whether this variation was purposeful. Regardless, it is unlikely that the same four bacterial species would similarly benefit host taxa spanning obligate carnivores (cats), omnivores (dogs), herbivores (equids and ruminants), and “multi-species” [5, 74–77]. This not only reflects on the probiotics specifically labeled for wildlife but also highlights potential concerns over the off-label use of probiotics not marketed for wildlife species. Additionally, we were not able to assess the viability of probiotic taxa in this study. Previous studies have demonstrated minimal and/or mislabeled viability in commercial probiotics marketed for animals [78, 79], adding to the potential limitations of commercial probiotics. Our results suggest that these probiotics were not tailored to the wildlife species for which they were being used, with future studies needed to assess probiotic quality, viability, and relevance across target host species.

Although contamination is difficult to assess, our stringent criteria—including use of laboratory controls, analytic decontamination, and four criteria for contaminant assignment, such as being in a genus not labeled on the probiotic—provided a robust foundation for identifying potential contaminants. Certain potential contaminants were only present in probiotics from the same company, such as the abundant *Streptococcus* contaminants. A *Streptococcus* species (*S. oralis*) was previously found as a contaminant in probiotic batches used in a neonatal intensive care units, with the manufacturer confirming contamination [80]. In an analysis of 92 animal-use probiotics from China, 33.7% of products were contaminated with potentially life-threatening pathogens [81]. In this study, four probiotics had no potential contaminants, two of which were marketed for humans, suggesting potentially stricter manufacturing practices for human-targeted probiotics. However, in a meta-analysis of human commercial probiotics, contamination was found in probiotics regulated as drugs (~18% of 38 products) and as dietary supplements (~15% of 114) [82]. Contamination of commercial probiotics has caused at least one human fatality from GI mucormycosis in a premature neonate [83]. We did not assess multiple batches of probiotics nor identify the stage at which the contaminants may have entered the probiotic product, and so we cannot speculate on whether these contaminants are consistent across probiotic production. Nonetheless, whether contamination of commercial probiotics could lead to health concerns in non-model animals has not been tested yet warrants consideration.

We found further evidence of taxonomic mislabeling, an increasing concern for microbial studies and industries. The expansion of DNA-based microbial research has accelerated changes to bacterial taxonomy and nomenclature [84–86]. A study of commercial cultures used for probiotic formulation found that 28% of 213 were misidentified at the genus or species level [87]. A 2024 study using metagenomic sequencing of US commercial probiotics found that ~33% of probiotics had label inaccuracies [88]. Here, we found that a taxon labeled as the obsolete *Streptococcus faecium* was likely *Enterococcus faecium*, which was reclassified over 40 years ago [89]. Given the commercial nature of probiotics and minimal regulations for dietary supplements, using appropriate nomenclature is critical to ensure accurate interpretation of probiotic content [85, 90].

When examining the presence of probiotic taxa in wildlife gut microbiomes, we found that most probiotic taxa did not match wildlife community members. *Enterococcus* sequences, however, were abundant in some animal hosts. *Enterococcus* members, such as *E. faecium* and *E. faecalis*, are abundant in the gut microbiomes of humans and some wild and domestic animals [91]. There are, however, important caveats to our comparison with the Song *et al*. dataset, which are mentioned in the Methods [55]. Namely, the limitation of only being able match the first 100bp of sequences hampered more fine-grained taxonomic matching and identification. In addition, we could not assess species or strain specificity with the amplicon data, which likely would have reflected varying *Enterococcus* taxa across wildlife species. These results highlight the discrepancy between bacteria used for commercial probiotics and those present in animal microbiomes.

We found limited evidence of probiotic colonization in BFF gut microbiomes. Most of the *Lactobacillus* and *Bifidobacterium* species labeled on the probiotic were rare or absent in ferret microbiomes and showed minimal variation across experimental group and over time. When examining bacterial association networks, the probiotics may have increased the network centrality of *Lactobacillus* in PBX animals during probiotic treatment, but that level of centrality was still similar to control animals. Two *Bifidobacterium* ASVs in the PBX group showed a short-lived increase in network centrality following probiotic treatment. These ASVs were identified as *B. pseudolongum*, a species that was abundant in ferrets but was not on the probiotic label. *Bifidobacterium* show species- and strain-level specificity in their interactions with the host’s immune system and resident microbiome [92, 93]. In a study of the probiotic *B. longum* AH1206—a native gut bacterium in humans—only 30% of human subjects showed persistent engraftment, which was dictated by the abundance of other genetically similar *Bifidobacterium* members. Greater prevalence of other *Bifidobacterium* strains was correlated with lower probiotic engraftment of *B. longum*. Our pattern of *Bifidobacterium* network centrality may reflect interactions between the closely related *Bifidobacterium* taxa. Given that *B. pseudolongum* is of animal origin whereas *B. longum*—one of the labeled probiotic taxa—is of human origin, our data may reflect increased activity in the ferret-native *B. pseudolongum* in response to non-native *B. longum*. These patterns demonstrate that microbial dynamics of host-specific native gut microbes may shape response to probiotic treatment.

In contrast to the rarity of *Lactobacillus* and *Bifidobacterium* species, *C. perfringens* was abundant in ferrets, reiterating previous findings [53] and indicating that certain strains or phenotypes are not acutely pathogenic. Although *C. perfringens* abundance did not respond to probiotic treatment, *C. perfringens* network centrality was significantly lower in the PBX group compared to the CON group during probiotic treatment. Given that we did not find a significant decrease in *C. perfringens* abundance, this shift in network centrality likely reflected changes in abundance and persistence of other bacterial taxa [95, 96]. In dairy calves, daily probiotic treatment with *Lactobacillus, Propionibacterium*, and *Bacillus* reduced incidences of severe *C. perfringens*-induced diarrhea and improved calf survival [94]. In contrast, a randomized, placebo-controlled study of neonatal foals reported that a 3-week probiotic treatment increased diarrheal symptoms and did not decrease *C. perfringens* shedding [95]. Our study was testing the influence of prophylactic probiotics in clinically healthy ferrets without active *C. perfringens* infection. Further research is needed to better characterize the genomes of ferret-associated *C. perfringens* and identify probiotic strains with antagonistic effects on pathogenic *C. perfringens* in ferrets.

Probiotic treatment showed minimal impact on microbial function in BFF gut microbiomes. Octanoyl-acyl carrier protein biosynthesis and myristate biosynthesis pathways, are components of fatty acid biosynthesis in bacteria [96–98]. These pathways were upregulated in CON animals compared to PBX animals, suggesting that the probiotic treatment may have had a stabilizing effect on microbiome function. Nevertheless, we found no evidence of upregulation of microbial functions in response to probiotic treatment. Previous research has shown that probiotics may exert influence on non-microbial aspects of host health, including via immune modulation and/or epigenetic modification [5]. A recent study of topical probiotic application in frogs demonstrated that short-term probiotic colonization occurred in tandem with altered immune gene expression, with specific probiotic taxa linked to distinct immune modulation [99]. Although beyond the scope of this study, future research on the influence of probiotics should consider such non-microbial influences and assess physiological or health outcomes in parallel with microbiome assessments. Pairing microbial data with host health metrics (e.g. inflammatory markers, immune responses) will provide critical insight into the role of probiotics in host–microbe interactions, which can lead to actionable recommendations for wildlife care. Moreover, we acknowledge that our 1-week probiotic treatment—which aligned with our goal of assessing existing probiotic practices and manufacturer guidelines—was a relatively short period and may have truncated potential effects on microbial function.

Overall, we identified numerous scientific and practical concerns for commercial probiotics marketed for animals, with little support for use in wildlife. We emphasize the need for greater integration of empirical, microbial ecology research into probiotic development. We further encourage researchers to study such questions across multiple, larger populations of wildlife, to increase sample sizes and examine microbial dynamics under different environments. Robust guidelines for future research and application of probiotics in wildlife species have been proposed, with a focus on treating high-priority wildlife pathogens [5]. Generation of high-throughput genomic data, paired with advances in data mining techniques, provide promising avenues for future identification of wildlife-specific probiotics. There are, however, innumerable settings in which probiotics are used to treat animals, necessitating a balance between optimization and accessibility. For most facilities that manage the health and conservation of numerous wildlife species (e.g. zoos), identifying, isolating, and producing host-specific probiotics is infeasible. In these cases, alternative microbial therapies that are adaptable to specific hosts (e.g. prebiotics or fecal microbiota transplants) may currently provide greater efficacy compared to commercial probiotics [7].

## Supplementary Material

ycag036_Supplemental_File

## Data Availability

Raw sequencing data for this project have been deposited in NCBI’s Sequence Read Archive under BioProjects PRJNA1330301 (16S probiotic data: https://www.ncbi.nlm.nih.gov/bioproject/PRJNA1330301), PRJNA1330255 (16S ferret data; https://www.ncbi.nlm.nih.gov/bioproject/PRJNA1330255), and PRJNA1337465 (metagenomic ferret data; https://www.ncbi.nlm.nih.gov/bioproject/PRJNA1337465). Additional files and analysis scripts are available at Open Science Framework (https://osf.io/dh92x/?view_only=e2ad4be181d349a49cdee3bd7e971e03).

## Disclaimers

Official contribution of the National Institute of Standards and Technology; not subject to copyright in the United States. All opinions expressed in this report are the authors’ and do not necessarily reflect the policies and views of the National Institute of Standards and Technology (NIST) or affiliated venues. Certain pieces of commercial equipment, instruments, or materials are identified in this paper to specify the experimental procedure adequately. Such identification is not intended to imply recommendation or endorsement by NIST, nor is it intended to imply that the materials or equipment identified are necessarily the best available for the purpose.
