# Supplementary material for: How “pro” are probiotics for wildlife species? Novel data, lack of evidence, and future directions
Source: ISME Commun. 2026 Feb 28;6(1):ycag036. doi: 10.1093/ismeco/ycag036 (PMC13077292; doi:10.1093/ismeco/ycag036)
Supplement: ycag036_Supplemental_File [file ycag036_supplemental_file.docx]

**How “pro” are probiotics for the care of wildlife animal species? Novel data, lack of evidence, and future directions**

**Supplementary Information**

***Supplementary Methods***

*Probiotic identification and processing*

Probiotics were all over-the-counter (no prescription required) and purchased directly from the manufacturer. Probiotics were not opened before extraction and all manufacturer seals were intact. Probiotics were only unsealed once in a sterile biosafety cabinet in a dedicated extraction lab while using personal protective equipment and sterile instruments to prevent contamination. The probiotics were extracted in random order, in quadruplicate (i.e., four separate preparations of each probiotic), and all probiotics were extracted, prepared, and sequenced in one batch. During extraction we took extra steps to prevent cross-contamination, including blank columns to separate wells across our plate.

*Ferret experimental design and sample collection – BFF husbandry*

BFFs were housed individually in indoor enclosures of metal and plastic substrates with ALPHA-dri bedding. Ferrets were fed commercial carnivore diets (e.g., Milliken meats Toronto Zoo blend) and whole prey (processed or live hamsters and rats). At FCC, ferrets are fed once per day the following diets:

1. 5 days per week: processed carnivore diet (Toronto Small Carnivore), generally 70g per female and 80g per male. The exact amount is adjusted based on body condition and breeding status.
2. 2 days per week: rat or hamster carcasses of the same gram amounts listed above.

*16S amplicon bioinformatics – filtering and spurious ASV assessment*

ASVs identified as non-bacterial (Archaea, chloroplasts, and mitochondria) and those not assigned to Bacteria at Kingdom level were removed. To reduce spurious ASVs, we omitted ASVs with total read counts of < 10 and ASVs found in < 2 samples. To further assess the presence of spurious ASVs, we examined our extraction and PCR positive controls. The controls harbored no contaminants, and we identified eight bacterial genera, which matched those reported by the control manufacturer (ZymoBIOMICS). Among these genera, five were represented by two ASVs whereas the other three were represented by a single ASV.

*Preparation and sequencing of probiotic and ferret samples – 16S library preparation*

For 16S libraries, we included negative (type 1 ultrapure water) and positive (ZymoBIOMICS microbial DNA standard; Zymo, Irvine, CA, USA; Catalog No. D6305) PCR controls. PCRs were run in duplicate and products were visualized using gel electrophoresis. We cleaned the PCR libraries (Apollo 324 System; IntegenX Inc., Pleasanton, CA, USA), quantified using qPCR (KAPA Library Quantification Kit for Illumina platforms, Roche Molecular Systems), pooled in equimolar ratios, and sequenced on an Illumina MiSeq run (2 x 300 bp paired-end reads).

*Preparation and sequencing of probiotic and ferret samples – shotgun library preparation*

Whole genomic shotgun (WGS) libraries were prepared using Watchmaker Genomics DNA library preparation kits following the manufacturers protocol (Watchmaker Genomics, Boulder, CO). Briefly, DNA was simultaneously fragmented, end-repaired, and treated to create a single A-base overhang on each end. Following an SPRI bead-based cleanup, the DNA fragments were ligated using IDT xGen Unique Dual Indexing primers. The prepared libraries were quantified (Qubit, Life Technologies, Grand Island, NY) and pooled at equimolar concentrations. The pool was quantified via qPCR (NEBNext Library Quant Kit for Illumina, New England Biolabs, Ipswich, MA) and sequenced on an Illumina NovaSeq X Plus instrument using the 25B flowcell and paired-end 150 bp reads.

*Statistics – Network construction*

Network hubs were nodes with eigenvector centrality values above the 95 % quantile of all eigenvector centralities in the network. Clusters are identified via greedy modularity optimization. To additionally assess association during probiotic treatment (W3), we generated a network of ASVs that were differentially correlated between the two groups, identified using Pearson’s correlations and Fisher’s z-test, with adjustments for local false discovery rate.

***Supplementary Results***

*Probiotic treatment and bacterial association networks in ferret microbiomes – network characteristics*

For the association networks that included all samples, qualitatively, the CON network had a slightly higher clustering coefficient (CON = 0.252, PBX = 0.225) and greater modularity (CON = 0.462, PBX = 0.410), potentially indicating more distinct clustering across bacterial taxa. However, the PBX network had slightly greater edge density (CON = 0.078, PBX = 0.085), suggesting greater interconnectivity between nodes. In bacterial networks of the probiotic treatment period (W3), the PBX network had slightly greater clustering coefficient (Figure 5; CON: 0.211, PBX: 0.287) and network modularity (CON: 0.536, PBX: 0.571) compared to the CON network, potentially indicating that probiotic treatment facilitated greater density and modularity of bacterial cluster associations.

***Supplementary Discussion***

We found evidence of taxonomic mislabeling, an increasing concern for microbial studies and industries. The expansion of DNA-based microbial research has accelerated changes to bacterial taxonomy and nomenclature (77–79). A study of commercial cultures used for probiotic formulation found that 28% of 213 were misidentified at the genus or species level (80). A 2024 study using metagenomic sequencing of U.S. commercial probiotics found that ~33% of probiotics had label inaccuracies (81). Here, a taxon labeled as the obsolete *Streptococcus faecium* was likely *Enterococcus faecium,* which was reclassified over 40 years ago (82). Given the commercial nature of probiotics and minimal regulations for dietary supplements, using appropriate nomenclature is critical to ensure accurate interpretation of probiotic content (78, 83).

**Supplementary Tables**

**Table S1.** Metadata for the black-footed ferrets included in the probiotic treatment experiment. CON = control, PBX = probiotic treated.

| Animal ID | Sex | Cage | Experimental group |
| --- | --- | --- | --- |
| 10303 | F | 2B3 | CON |
| 10155 | F | 2B5 | CON |
| 9837 | M | 2C1 | CON |
| 9672 | F | 2C2 | CON |
| 9781 | F | 2C3 | CON |
| 9644 | M | 2C4 | CON |
| 9757 | F | 2C5 | CON |
| 9443 | M | 2C6 | CON |
| 9435 | F | 2C7 | CON |
| 10422 | M | 2D2 | CON |
| 9872 | M | 2D4 | CON |
| 9705 | M | 2D6 | CON |
| 10402 | F | 2A1 | PBX |
| 9868 | M | 2A2 | PBX |
| 9869 | F | 2A3 | PBX |
| 10018 | F | 2A4 | PBX |
| 10437 | M | 2A5 | PBX |
| 9727 | F | 2A6 | PBX |
| 9614 | M | 2A7 | PBX |
| 10080 | F | 2B1 | PBX |
| 9373 | M | 2B2 | PBX |
| 9652 | M | 2B4 | PBX |
| 9836 | M | 2B6 | PBX |
| 9795 | F | 2B7 | PBX |

**Table S2.** Full list of ingredients in the probiotics used in the black-footed ferret treatment experiment.

| **Ingedients:** |
| --- |
| maltodextrin |
| fructooligosaccharides |
| dried Lactobacillus acidophilus fermentation product |
| dried Lactobacillus plantarum fermentation product |
| silica |
| dried Lactobacillus casei fermentation product |
| dried Lactobacillus rhamnosus fermentation product |
| dried Bifidobacterium |
| dried Lactobacillus reuteri fermentation product |
| bifidum fermentation product |
| dried Bifidobacterium longum fermentation product |
| dried Lactobacillus bulgaricus fermentation product |
| **Analysis per serving (1 gram)** |
| Moisture (max): 5.10% |
| FOS:Fructooligosaccharides: 200mg |
| Total microorganisms: 10 Billion CFU/gram |
| *Lactobacillus acidophilus* |
| *Lactobacillus plantarum* |
| *Lactobacillus casei* |
| *Lactobacillus rhamnosus* |
| *Bifidobacterium bifidum* |
| *Bifidobacterium longum* |
| *Lactobacillus bulgaricus* |
| *Lactobacillus reuteri* |

**Table S3**. Representative sequences, Feature ID, taxonomic assignment, and the number of host species for the sequences that matched between the commercial probiotics and sequences generated in either the Song et al dataset or the novel dataset from cheetahs, Coquerel’s sifaka, giant pandas, and red pandas.

| Dataset | Representative sequence | Feature ID | Taxon | # of host species |
| --- | --- | --- | --- | --- |
| Song et al | TACGTAGGTGGCAAGCGTTATCCGGATTTATTGGGCGTAAAGCGAGCGCAGGCGGTTTTTTAAGTCTGATGTGAAAGCCCTCGGCTTAACCGAGGAAGTG | 270383 | k Bacteria;  p Firmicutes;  c Bacilli;  o Lactobacillales;  f Lactobacillaceae;  g Lactobacillus;  s zeae | 9 |
|  | TACGTAGGTGGCAAGCGTTGTCCGGATTTATTGGGCGTAAAGCGAGCGCAGGCGGAAGAATAAGTCTGATGTGAAAGCCCTCGGCTTAACCGAGGAACTG | 578653 | k Bacteria;  p Firmicutes;  c Bacilli;  o Lactobacillales;  f Lactobacillaceae;  g Lactobacillus | 104 |
|  | TACGTAGGTGGCAAGCGTTGTCCGGATTTATTGGGCGTAAAGCGAGCGCAGGCGGTTTTTTAAGTCTGATGTGAAAGCCTTCGGCTCAACCGAAGAAGTG | 583868 | k Bacteria;  p Firmicutes;  c Bacilli;  o Lactobacillales;  f Lactobacillaceae; | 59 |
|  | TACGTAGGTGGCAAGCGTTATCCGGATTTATTGGGCGTAAAGCGAGCGCAGGCGGTCTTTTAAGTCTAATGTGAAAGCCTTCGGCTCAACCGAAGAAGTG | 632309 | k Bacteria;  p Firmicutes;  c Bacilli;  o Lactobacillales;  f Lactobacillaceae;  g Pediococcus | 92 |
|  | TACGTAGGTGGCAAGCGTTATCCGGATTTATTGGGCGTAAAGCGAGCGCAGGCGGTTTTTTAAGTCTGATGTGAAAGCCCTCGGCTTAACCGAGGAAGCG | 767952 | k Bacteria;  p Firmicutes;  c Bacilli;  o Lactobacillales;  f Lactobacillaceae;  g Lactobacillus;  s zeae | 8 |
|  | TACGTAGGGTGCGAGCGTTATCCGGATTTATTGGGCGTAAAGGGCTCGTAGGCGGTTCGTCGCGTCCGGTGTGAAAGTCCATCGCCTAACGGTGGATCTG | 4331147 | k Bacteria;  p Actinobacteria;  c Actinobacteria;  o Bifidobacteriales;  f Bifidobacteriaceae;  g Bifidobacterium;  s animalis | 18 |
|  | TACGTAGGTGGCAAGCGTTGTCCGGATTTATTGGGCGTAAAGCGAGCGCAGGCGGTTTCTTAAGTCTGATGTGAAAGCCCCCGGCTCAACCGGGGAGGGT | 4359590 | k Bacteria;  p Firmicutes;  c Bacilli;  o Lactobacillales;  f Enterococcaceae;  g Enterococcus | 605 |
|  | TACGTAGGTGGCAAGCGTTATCCGGATTTATTGGGCGTAAAGAGAGTGCAGGCGGTTTTCTAAGTCTGATGTGAAAGCCTTCGGCTTAACCGGAGAAGTG | 4439743 | k Bacteria;  p Firmicutes;  c Bacilli;  o Lactobacillales;  f Lactobacillaceae;  g Lactobacillus | 8 |

**Table S4.** Results of hierarchical generalized additive models of longitudinal variation in bacterial alpha diversity in black-footed ferret gut microbiomes.

|  |  | edf | Ref.df | F | p-value |
| --- | --- | --- | --- | --- | --- |
| Shannon | s(day):groupcontrol | 2.061 | 2.546 | 2.619 | 0.082 |
| R^2^ = 0.543 | s(day):grouptreat | 1.000 | 1.000 | 0.357 | 0.551 |
|  | s(animal) | 21.440 | 23.000 | 13.695 | 2.00E-16 |
|  | s(group) | 1.85E-05 | 1.000 | 0.000 | 0.673 |
| Observed | s(days_since_pbx):groupcontrol | 4.859 | 5.851 | 5.420 | 0.000 |
| R^2^ = 0.486 | s(days_since_pbx):grouptreat | 4.516 | 5.442 | 1.949 | 0.082 |
|  | s(animal) | 20.740 | 23.000 | 9.318 | 2.00E-16 |
|  | s(group) | 0.001 | 1.000 | 0.000 | 0.887 |
| Faith’s phylogenetic diversity | s(days_since_pbx):groupcontrol | 1.000 | 1.000 | 4.291 | 0.039 |
| R^2^ = 0.328 | s(days_since_pbx):grouptreat | 1.001 | 1.002 | 0.447 | 0.504 |
|  | s(animal) | 19.390 | 23.000 | 5.560 | 2.00E-16 |
|  | s(group) | 4.47E-05 | 1.000 | 0.000 | 0.739 |

**Table S5.** Results of hierarchical generalized additive models of longitudinal variation in center-log ratio transformed abundances of ASVs identified as *Bifidobacterium*, *Lactobacillus*, or *Clostridium perfringens* in black-footed ferret gut microbiomes.

| Taxon | Variable | edf | Ref.df | F | p-value |
| --- | --- | --- | --- | --- | --- |
| Bifidobacterium ASV494 | CON:by day | 3.634 | 4.416 | 3.627 | 0.004 |
| R^2^ = 0.243 | PBX:by day | 2.712 | 3.324 | 4.368 | 0.004 |
|  | animal | 16.530 | 23.000 | 2.454 | 0.000 |
|  | group | 0.000 | 1.000 | 0.000 | 0.825 |
| Bifidobacterium ASV495 | CON:by day | 5.590 | 6.681 | 2.249 | 0.030 |
| R^2^ = 0.0547 | PBX:by day | 1.000 | 1.001 | 0.194 | 0.660 |
|  | animal | 0.001 | 23.000 | 0.000 | 0.995 |
|  | group | 0.228 | 1.000 | 0.295 | 0.256 |
| Bifidobacterium ASV496 | CON:by day | 1.118 | 1.225 | 4.877 | 0.018 |
| R^2^ = 0.0174 | PBX:by day | 1.000 | 1.000 | 0.138 | 0.711 |
|  | animal | 0.000 | 23.000 | 0.000 | 0.925 |
|  | group | 0.000 | 1.000 | 0.000 | 0.959 |
| Bifidobacterium;  Bifidobacterium animalis ASV497 | CON:by day | 1.000 | 1.000 | 1.241 | 0.266 |
| R^2^ = 0.0313 | PBX:by day | 1.001 | 1.002 | 0.056 | 0.814 |
|  | animal | 6.241 | 23.000 | 0.367 | 0.144 |
|  | group | 0.359 | 1.000 | 0.735 | 0.213 |
| Bifidobacterium ASV498 | CON:by day | 1.545 | 1.900 | 0.435 | 0.691 |
| R^2^ = 0.148 | PBX:by day | 2.579 | 3.167 | 1.312 | 0.237 |
|  | animal | 14.930 | 23.000 | 1.832 | 0.000 |
|  | group | 0.000 | 1.000 | 0.000 | 0.567 |
| Bifidobacterium;  Bifidobacterium bifidum ASV502 | CON:by day | 1.000 | 1.000 | 0.000 | 1.000 |
| R^2^ = 0.0222 | PBX:by day | 3.436 | 4.182 | 1.794 | 0.115 |
|  | animal | 0.000 | 23.000 | 0.000 | 1.000 |
|  | group | 0.000 | 1.000 | 0.000 | 0.964 |
| Bifidobacterium | CON:by day | 1.000 | 1.000 | 3.201 | 0.075 |
| R^2^ = 0.0949 | PBX:by day | 1.392 | 1.683 | 0.843 | 0.522 |
|  | animal | 12.070 | 23.000 | 1.099 | 0.003 |
|  | group | 0.000 | 1.000 | 0.000 | 0.450 |
| Lactobacillus ASV175 | CON:by day | 1.001 | 1.002 | 0.118 | 0.733 |
| R^2^ = -0.00016 | PBX:by day | 1.000 | 1.000 | 0.142 | 0.707 |
|  | animal | 1.580 | 23.000 | 0.074 | 0.370 |
|  | group | 0.000 | 1.000 | 0.000 | 0.454 |
| Lactobacillus ASV177 | CON:by day | 1.002 | 1.003 | 0.258 | 0.612 |
| R^2^ = -0.00283 | PBX:by day | 1.000 | 1.000 | 0.000 | 1.000 |
|  | animal | 0.950 | 23.000 | 0.043 | 0.412 |
|  | group | 0.000 | 1.000 | 0.000 | 0.998 |
| Lactobacillus ASV178 | CON:by day | 1.000 | 1.000 | 0.026 | 0.873 |
| R^2^ = 0.0242 | PBX:by day | 1.000 | 1.000 | 8.285 | 0.004 |
|  | animal | 0.300 | 23.000 | 0.013 | 0.447 |
|  | group | 0.000 | 1.000 | 0.000 | 0.994 |
| Lactobacillus ASV179 | CON:by day | 1.000 | 1.000 | 0.356 | 0.551 |
| R^2^ = 0.0106 | PBX:by day | 1.092 | 1.177 | 0.009 | 0.971 |
|  | animal | 3.300 | 23.000 | 0.169 | 0.267 |
|  | group | 0.288 | 1.000 | 0.446 | 0.237 |
| Lactobacillus ASV182 | CON:by day | 1.199 | 1.370 | 0.332 | 0.549 |
| R^2^ = 0.0157 | PBX:by day | 2.086 | 2.584 | 1.574 | 0.288 |
|  | animal | 1.678 | 23.000 | 0.079 | 0.370 |
|  | group | 0.000 | 1.000 | 0.000 | 0.830 |
| Lactobacillus ASV183 | CON:by day | 1.166 | 1.312 | 0.214 | 0.829 |
| R^2^ = 0.000391 | PBX:by day | 1.000 | 1.000 | 0.683 | 0.409 |
|  | animal | 0.099 | 23.000 | 0.004 | 0.461 |
|  | group | 0.501 | 1.000 | 1.008 | 0.158 |
| Lactobacillus ASV187 | CON:by day | 2.282 | 2.810 | 2.300 | 0.109 |
| R^2^ = 0.0173 | PBX:by day | 1.270 | 1.491 | 0.107 | 0.781 |
|  | animal | 0.002 | 23.000 | 0.000 | 0.631 |
|  | group | 0.000 | 1.000 | 0.000 | 0.709 |
| Lactobacillus ASV188 | CON:by day | 1.628 | 2.012 | 0.868 | 0.420 |
| R^2^ = 0.0222 | PBX:by day | 1.635 | 2.025 | 0.620 | 0.525 |
|  | animal | 0.000 | 23.000 | 0.000 | 0.872 |
|  | group | 0.832 | 1.000 | 4.937 | 0.016 |
| Lactobacillus ASV189 | CON:by day | 1.000 | 1.000 | 0.000 | 1.000 |
| R^2^ = 0.00658 | PBX:by day | 2.281 | 2.817 | 1.482 | 0.320 |
|  | animal | 0.000 | 23.000 | 0.000 | 1.000 |
|  | group | 0.000 | 1.000 | 0.000 | 0.987 |
| Lactobacillus ASV191 | CON:by day | 1.000 | 1.000 | 0.001 | 0.981 |
| R^2^ = -0.00733 | PBX:by day | 1.000 | 1.000 | 0.057 | 0.812 |
|  | animal | 0.001 | 23.000 | 0.000 | 0.555 |
|  | group | 0.000 | 1.000 | 0.000 | 0.825 |
| Lactobacillus ASV195 | CON:by day | 1.760 | 2.181 | 1.485 | 0.198 |
| R^2^ = 0.0135 | PBX:by day | 1.000 | 1.000 | 0.693 | 0.406 |
|  | animal | 0.226 | 23.000 | 0.010 | 0.452 |
|  | group | 0.496 | 1.000 | 0.993 | 0.160 |
| Lactobacillus ASV196 | CON:by day | 1.000 | 1.000 | 8.588 | 0.004 |
| R^2^ = 0.0318 | PBX:by day | 1.000 | 1.000 | 0.000 | 1.000 |
|  | animal | 2.044 | 23.000 | 0.098 | 0.344 |
|  | group | 0.000 | 1.000 | 0.000 | 0.993 |
| Lactobacillus ASV199 | CON:by day | 1.000 | 1.000 | 0.003 | 0.956 |
| R^2^ = 0.034 | PBX:by day | 3.543 | 4.309 | 2.427 | 0.040 |
|  | animal | 0.450 | 23.000 | 0.020 | 0.438 |
|  | group | 0.000 | 1.000 | 0.000 | 0.990 |
| Lactobacillus ASV200 | CON:by day | 1.000 | 1.001 | 0.114 | 0.737 |
| R^2^ = 0.0109 | PBX:by day | 1.000 | 1.000 | 0.265 | 0.607 |
|  | animal | 3.462 | 23.000 | 0.179 | 0.257 |
|  | group | 0.231 | 1.000 | 0.326 | 0.255 |
| Lactobacillus ASV201 | CON:by day | 1.000 | 1.000 | 0.003 | 0.956 |
| R^2^ = 0.0279 | PBX:by day | 3.214 | 3.919 | 1.828 | 0.095 |
|  | animal | 1.314 | 23.000 | 0.061 | 0.385 |
|  | group | 0.000 | 1.000 | 0.000 | 0.848 |
| Lactobacillus ASV203 | CON:by day | 1.000 | 1.001 | 0.000 | 1.000 |
| R^2^ = -0.00399 | PBX:by day | 1.000 | 1.000 | 0.027 | 0.870 |
|  | animal | 0.878 | 23.000 | 0.040 | 0.418 |
|  | group | 0.000 | 1.000 | 0.000 | 0.994 |
| Lactobacillus;  Lactobacillus brevis ASV198 | CON:by day | 1.000 | 1.001 | 0.128 | 0.721 |
| R^2^ = 0.0107 | PBX:by day | 1.000 | 1.000 | 1.324 | 0.251 |
|  | animal | 2.524 | 23.000 | 0.125 | 0.311 |
|  | group | 0.296 | 1.000 | 0.453 | 0.234 |
| Lactobacillus;  Lactobacillus fermentum ASV204 | CON:by day | 1.000 | 1.000 | 0.001 | 0.979 |
| R^2^ = -0.00336 | PBX:by day | 1.000 | 1.000 | 0.200 | 0.655 |
|  | animal | 0.873 | 23.000 | 0.039 | 0.416 |
|  | group | 0.000 | 1.000 | 0.000 | 0.918 |
| Lactobacillus;  Lactobacillus intestinalis ASV194 | CON:by day | 1.000 | 1.000 | 1.939 | 0.165 |
| R^2^ = 0.0544 | PBX:by day | 1.917 | 2.379 | 1.113 | 0.387 |
|  | animal | 8.354 | 23.000 | 0.569 | 0.051 |
|  | group | 0.001 | 1.000 | 0.001 | 0.339 |
| Lactobacillus;  Lactobacillus intestinalis ASV197 | CON:by day | 1.000 | 1.000 | 0.046 | 0.830 |
| R^2^ = 0.0158 | PBX:by day | 2.009 | 2.492 | 2.042 | 0.138 |
|  | animal | 0.000 | 23.000 | 0.000 | 0.600 |
|  | group | 0.577 | 1.000 | 1.365 | 0.125 |
| Lactobacillus;  Lactobacillus saerimneri ASV184 | CON:by day | 1.000 | 1.000 | 0.000 | 1.000 |
| R^2^ = -0.00658 | PBX:by day | 1.000 | 1.000 | 0.256 | 0.613 |
|  | animal | 0.000 | 23.000 | 0.000 | 1.000 |
|  | group | 0.000 | 1.000 | 0.000 | 0.998 |
| Lactobacillus | CON:by day | 1.000 | 1.001 | 2.448 | 0.119 |
| R^2^ = 0.0444 | PBX:by day | 1.000 | 1.000 | 0.288 | 0.592 |
|  | animal | 7.698 | 23.000 | 0.505 | 0.068 |
|  | group | 0.000 | 1.000 | 0.000 | 0.754 |
| Clostridium sensu stricto 1 ASV1 | CON:by day | 1.000 | 1.000 | 0.216 | 0.643 |
| R^2^ = -0.000344 | PBX:by day | 1.000 | 1.000 | 0.851 | 0.357 |
|  | animal | 0.000 | 23.000 | 0.000 | 0.507 |
|  | group | 0.458 | 1.000 | 0.844 | 0.176 |
| Clostridium sensu stricto 1 ASV2 | CON:by day | 1.000 | 1.000 | 0.776 | 0.379 |
| R^2^ = 0.0525 | PBX:by day | 1.000 | 1.000 | 2.460 | 0.118 |
|  | animal | 8.399 | 23.000 | 0.584 | 0.044 |
|  | group | 0.000 | 1.000 | 0.000 | 0.742 |
| Clostridium sensu stricto 1 ASV3 | CON:by day | 1.003 | 1.006 | 1.342 | 0.247 |
| R^2^ = -0.00243 | PBX:by day | 1.000 | 1.000 | 0.000 | 1.000 |
|  | animal | 0.001 | 23.000 | 0.000 | 0.758 |
|  | group | 0.000 | 1.000 | 0.000 | 0.996 |
| Clostridium sensu stricto 1 ASV553 | CON:by day | 2.271 | 2.795 | 1.687 | 0.171 |
| R^2^ = 0.104 | PBX:by day | 1.000 | 1.000 | 0.295 | 0.587 |
|  | animal | 12.360 | 23.000 | 1.190 | 0.002 |
|  | group | 0.000 | 1.000 | 0.000 | 0.404 |
| Clostridium sensu stricto 1 ASV554 | CON:by day | 3.325 | 4.047 | 3.445 | 0.009 |
| R^2^ = 0.426 | PBX:by day | 3.821 | 4.634 | 4.604 | 0.001 |
|  | animal | 20.270 | 23.000 | 7.089 | 0.000 |
|  | group | 0.000 | 1.000 | 0.000 | 0.748 |
| Clostridium sensu stricto 1 ASV555 | CON:by day | 1.000 | 1.000 | 0.166 | 0.684 |
| R^2^ = 0.00564 | PBX:by day | 1.984 | 2.462 | 1.332 | 0.264 |
|  | animal | 0.000 | 23.000 | 0.000 | 0.842 |
|  | group | 0.000 | 1.000 | 0.000 | 0.338 |
| Clostridium sensu stricto 1 ASV556 | CON:by day | 1.000 | 1.000 | 0.896 | 0.345 |
| R^2^ = 0.0641 | PBX:by day | 1.022 | 1.044 | 1.284 | 0.248 |
|  | animal | 9.444 | 23.000 | 0.652 | 0.063 |
|  | group | 0.636 | 1.000 | 3.894 | 0.099 |
| Clostridium sensu stricto 1 ASV557 | CON:by day | 1.000 | 1.000 | 0.000 | 1.000 |
| R^2^ = 0.00936 | PBX:by day | 2.513 | 3.091 | 1.265 | 0.261 |
|  | animal | 0.000 | 23.000 | 0.000 | 1.000 |
|  | group | 0.000 | 1.000 | 0.000 | 0.982 |
| Clostridium sensu stricto 1 ASV558 | CON:by day | 1.000 | 1.000 | 0.015 | 0.902 |
| R^2^ = 0.0393 | PBX:by day | 4.028 | 4.878 | 2.711 | 0.034 |
|  | animal | 0.000 | 23.000 | 0.000 | 0.972 |
|  | group | 0.000 | 1.000 | 0.000 | 0.474 |
| Clostridium sensu stricto 1 ASV559 | CON:by day | 1.000 | 1.000 | 0.283 | 0.595 |
| R^2^ = 0.0196 | PBX:by day | 2.355 | 2.904 | 2.132 | 0.082 |
|  | animal | 0.000 | 23.000 | 0.000 | 0.994 |
|  | group | 0.000 | 1.000 | 0.000 | 0.540 |
| Clostridium sensu stricto 1 ASV560 | CON:by day | 1.496 | 1.832 | 1.279 | 0.373 |
| R^2^ = 0.00168 | PBX:by day | 1.000 | 1.000 | 0.170 | 0.681 |
|  | animal | 0.000 | 23.000 | 0.000 | 0.814 |
|  | group | 0.215 | 1.000 | 0.274 | 0.260 |
| Clostridium sensu stricto 1 ASV561 | CON:by day | 1.000 | 1.000 | 0.566 | 0.452 |
| R^2^ = -0.00537 | PBX:by day | 1.000 | 1.000 | 0.007 | 0.934 |
|  | animal | 0.000 | 23.000 | 0.000 | 0.998 |
|  | group | 0.000 | 1.000 | 0.000 | 0.439 |
| Clostridium sensu stricto 1 ASV562 | CON:by day | 3.821 | 4.639 | 2.073 | 0.058 |
| R^2^ = 0.104 | PBX:by day | 1.000 | 1.000 | 0.115 | 0.735 |
|  | animal | 11.190 | 23.000 | 0.945 | 0.007 |
|  | group | 0.000 | 1.000 | 0.000 | 0.637 |
| Clostridium sensu stricto 1 ASV563 | CON:by day | 1.000 | 1.000 | 2.444 | 0.119 |
| R^2^ = 0.00474 | PBX:by day | 1.000 | 1.000 | 0.572 | 0.450 |
|  | animal | 0.253 | 23.000 | 0.011 | 0.452 |
|  | group | 0.000 | 1.000 | 0.000 | 0.331 |
| Clostridium sensu stricto 1 ASV565 | CON:by day | 1.000 | 1.000 | 0.433 | 0.511 |
| R^2^ = 0.0396 | PBX:by day | 3.080 | 3.760 | 1.942 | 0.134 |
|  | animal | 3.992 | 23.000 | 0.203 | 0.275 |
|  | group | 0.000 | 1.000 | 0.000 | 0.491 |
| Clostridium sensu stricto 1 ASV567 | CON:by day | 1.000 | 1.000 | 0.149 | 0.699 |
| R^2^ = 0.0287 | PBX:by day | 3.085 | 3.767 | 2.643 | 0.055 |
|  | animal | 0.000 | 23.000 | 0.000 | 1.000 |
|  | group | 0.000 | 1.000 | 0.000 | 0.530 |

**Table S6.** List of bacterial strains identified by shotgun metagenomics as *Bifidobacterium*, *Lactobacillus*, or *Clostridium perfringens* and their abundance metrics in the gut microbiomes of black-footed ferrets

| Bacterial species | strain | average | stdev | Min % | Max % |
| --- | --- | --- | --- | --- | --- |
| Lactobacillus acidophilus | SGB7044 | 0.021 | 0.131 | 0 | 1.067 |
| Lactobacillus intestinalis | SGB7055 | 0.251 | 0.994 | 0 | 5.334 |
| Lactobacillus johnsonii | SGB7041 | 0.360 | 2.276 | 0 | 18.942 |
| Bifidobacterium animalis | SGB100575 | 0.142 | 1.142 | 0 | 9.690 |
|  | SGB17278 | 0.001 | 0.006 | 0 | 0.046 |
| Bifidobacterium bifidum | SGB17256 | <0.001 | 0.001 | 0 | 0.004 |
| Bifidobacterium longum | SGB17248 | 0.003 | 0.026 | 0 | 0.222 |
| Bifidobacterium pseudocatenulatum | SGB17237 | <0.001 | 0.001 | 0 | 0.005 |
| Bifidobacterium pseudolongum | SGB17279 | 6.472 | 7.397 | 0 | 33.751 |
| Bifidobacterium pullorum | SGB17264 | 0.001 | 0.004 | 0 | 0.030 |
| Bifidobacterium thermophilum | SGB17273 | <0.001 | 0.001 | 0 | 0.006 |
| Clostridium perfringens | SGB6191 | 7.160 | 12.031 | 0 | 60.858 |

**Table S7.** Results of differential association analysis for Bifidobacterium associations that were significantly different between the CON and PBX networks during probiotic treatment.

| taxon 1 | taxon 2 | association - CON | association - PBX | p-value |
| --- | --- | --- | --- | --- |
| g:Bifidobacterium ASV494 | g:Allobaculum ASV135 | 0.783 | -0.031 | 0.001 |
| g:Bifidobacterium ASV494 | g:Allobaculum ASV136 | 0.768 | 0.137 | 0.006 |
| g:Bifidobacterium ASV494 | g:Enterococcus ASV147 | -0.540 | 0.220 | 0.010 |
| g:Bifidobacterium ASV498 | g:Ileibacterium ASV111 | 0.605 | -0.428 | <0.001 |
| g:Bifidobacterium ASV498 | g:Ileibacterium ASV121 | -0.111 | 0.615 | 0.010 |
| g:Bifidobacterium ASV498 | g:Ileibacterium ASV116 | -0.149 | 0.542 | 0.018 |
| g:Bifidobacterium ASV498 | g:Ileibacterium ASV119 | -0.107 | 0.557 | 0.022 |

**Supplementary Figures**

**
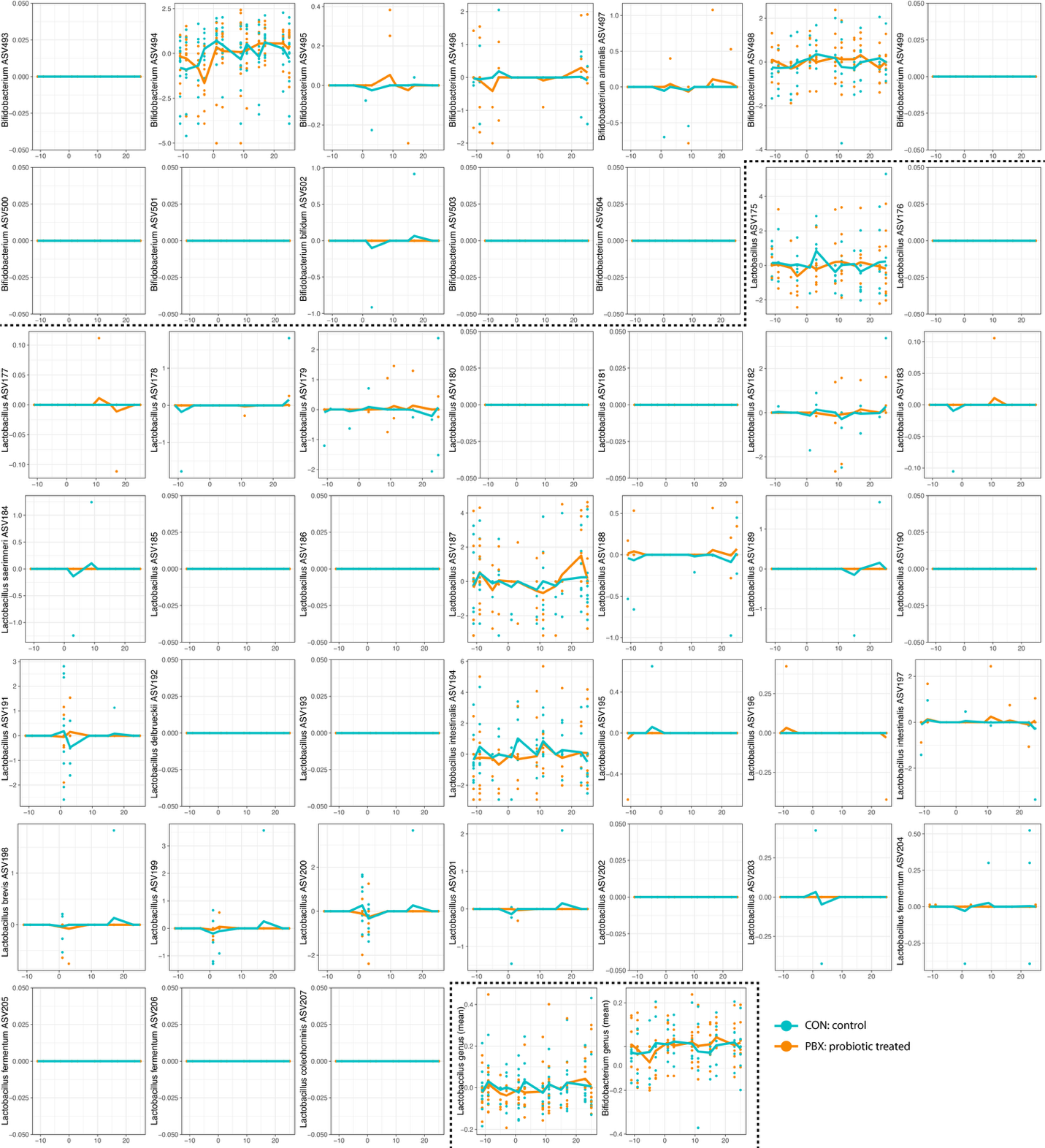
Figure S1.** Longitudinal variation in center-log ratio transformed abundances of *Bifidobacterium* and *Lactobacillus* ASVs in the gut microbiomes of control (CON) and probiotic-treated (PBX) black-footed ferrets.

**
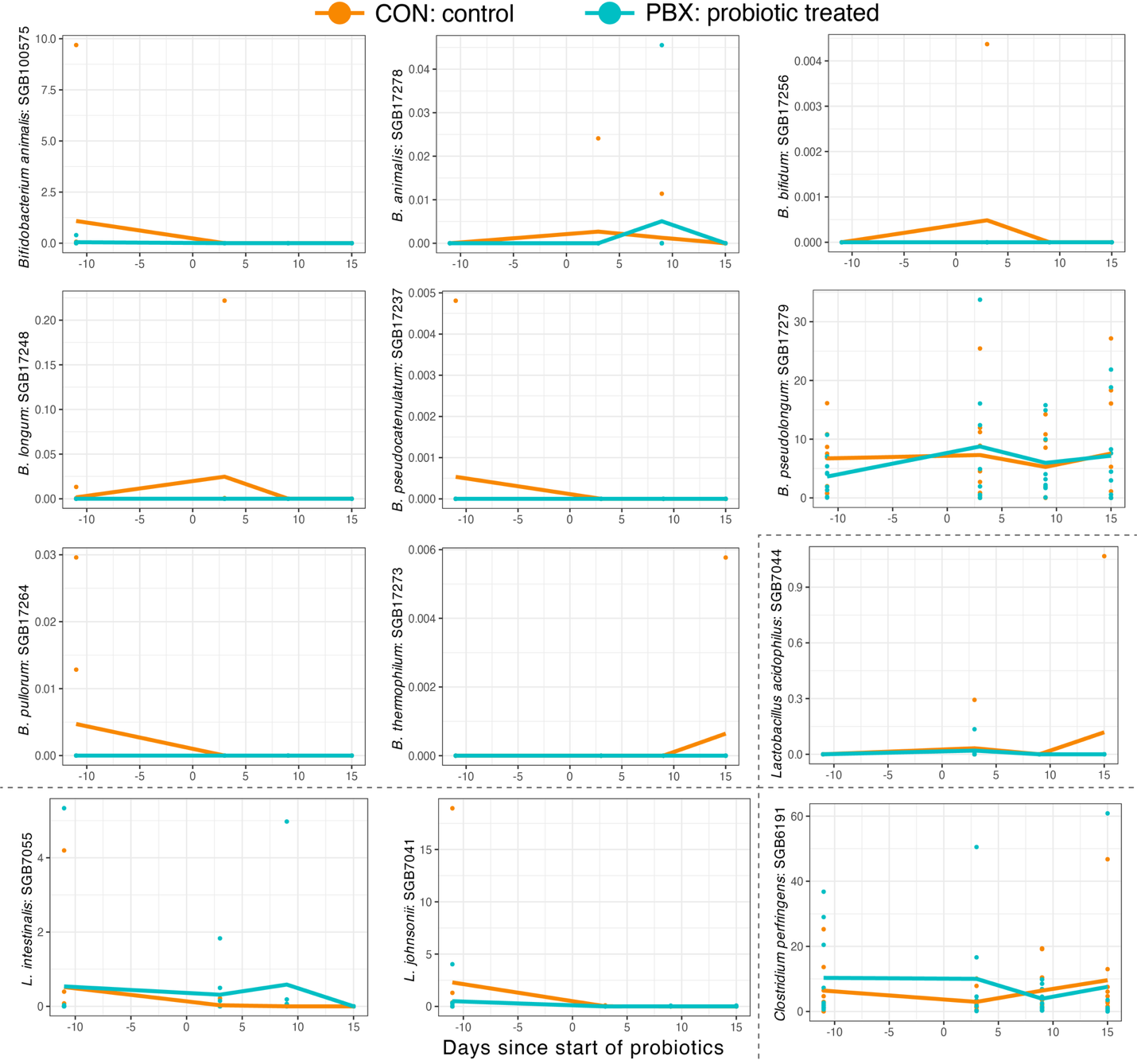
Figure S2.** Longitudinal variation in center-log ratio transformed abundances of *Bifidobacterium*, *Lactobacillus*, and *Clostridium perfringens* bacterial strains in the gut microbiomes of control (CON) and probiotic-treated (PBX) black-footed ferrets.

**
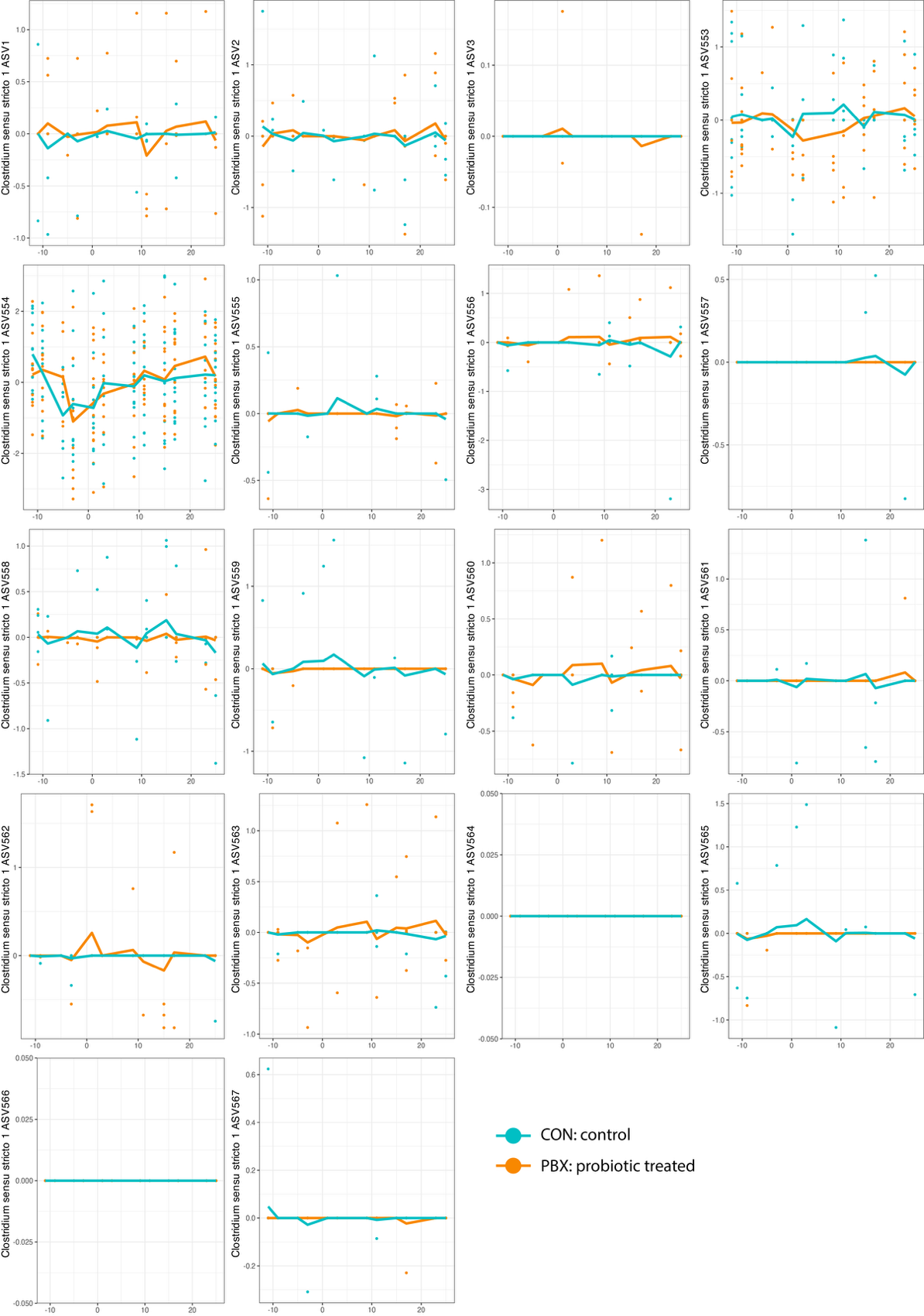
**

**Figure S3.** Longitudinal variation in center-log ratio transformed abundances of ASVs identified as *Clostridium perfringens* (genus *Clostridium sensu stricto 1*)in the gut microbiomes of control (CON) and probiotic-treated (PBX) black-footed ferrets.


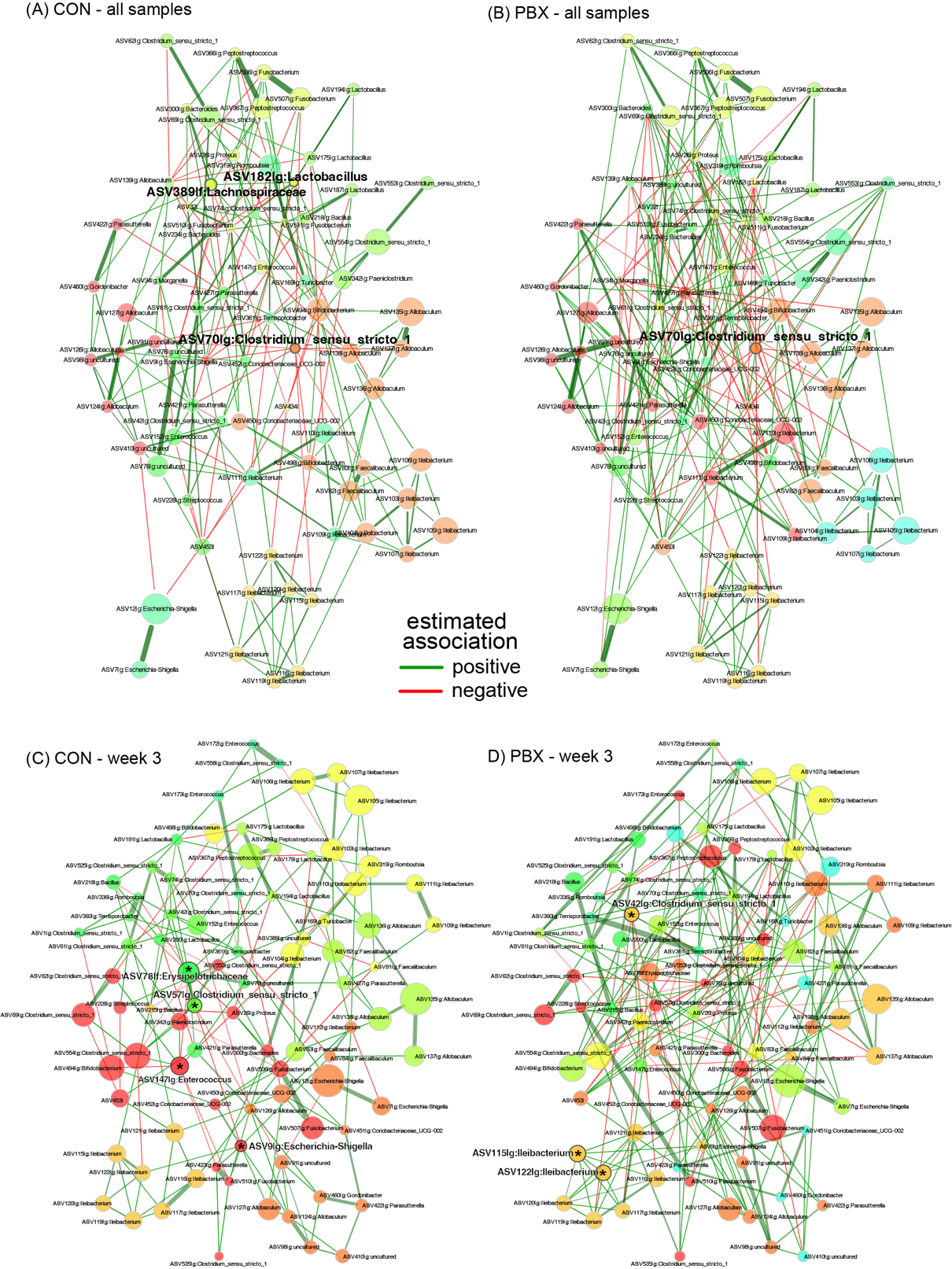


**Figure S4.** Association networks for the microbiomes of black-footed ferrets in (A,C) control (CON) or (B,D) probiotic-treated (PBX) experimental groups across all samples (A,B) and during the period of probiotic treatment (week 3; C,D). Networks are constructed with center log-ratio normalized abundances of amplicon sequence variants (ASVs) and nodes are scales to the taxon’s eigenvector centrality. Nodes are colored according to clusters calculated using greedy modularity optimization. Edge weights are scaled to (non-negative) similarities, with negative associations colored red. Hubs are nodes with an eigenvector centrality value above the empirical 95 % quantile of all eigenvector centralities in the network and are denoted in bold with asterisks.
